# Bronchial epithelia from adults and children: SARS-CoV-2 spread via syncytia formation and type III interferon infectivity restriction

**DOI:** 10.1073/pnas.2202370119

**Published:** 2022-06-24

**Authors:** Guillaume Beucher, Marie-Lise Blondot, Alexis Celle, Noémie Pied, Patricia Recordon-Pinson, Pauline Esteves, Muriel Faure, Mathieu Métifiot, Sabrina Lacomme, Denis Dacheux, Derrick R. Robinson, Gernot Längst, Fabien Beaufils, Marie-Edith Lafon, Patrick Berger, Marc Landry, Denis Malvy, Thomas Trian, Marie-Line Andreola, Harald Wodrich

**Affiliations:** ^a^UMR 5234, Microbiologie Fondamentale et Pathogénicité, CNRS, Université de Bordeaux, 33076 Bordeaux, France;; ^b^U1045, Centre de Recherche Cardio-thoracique de Bordeaux, INSERM, Université de Bordeaux, 33076 Bordeaux, France;; ^c^UMS 3420, Bordeaux Imaging Center (BIC), Université de Bordeaux, 33076 Bordeaux, France;; ^d^Bordeaux INP, Microbiologie Fondamentale et Pathogénicité, UMR 5234, 33076 Bordeaux, France;; ^e^Biochemistry Center Regensburg, Universität Regensburg, 93053 Regensburg, Germany;; ^f^Service de Pédiatrie Médicale, Centre Hospitalier Universitaire (CHU) de Bordeaux, 33076 Bordeaux, France;; ^g^Service de Virologie et l'unité de Surveillance Biologique, Centre Hospitalier Universitaire (CHU) de Bordeaux, 33076 Bordeaux, France;; ^h^Service d’Exploration Fonctionnelle Respiratoire, Centre d’Investigation Clinique (CIC) 1401, Centre Hospitalier Universitaire (CHU) de Bordeaux, 33076 Bordeaux, France;; ^i^UMR 5293, Institute of Neurodegenerative Diseases, Institut interdisciplinaire de neurosciences (IINS), CNRS, INSERM, Université de Bordeaux, 33076 Bordeaux, France;; ^j^Department for Infectious and Tropical Diseases, Centre Hospitalier Universitaire (CHU) de Bordeaux, 33076 Bordeaux, France

**Keywords:** SARS-CoV-2, bronchial epithelia, syncytia, interferon response, children

## Abstract

Severe acute respiratory syndrome coronavirus 2 (SARS-CoV-2) infections initiate in the bronchi of the upper respiratory tract and are able to disseminate to the lower respiratory tract, where infections can cause an acute respiratory distress syndrome with a high degree of mortality in elderly patients. We used reconstituted primary bronchial epithelia from adult and child donors to follow the SARS-CoV-2 infection dynamics. We show that, in epithelia from adult donors, infections initiate in multiciliated cells and spread within 24 to 48 h throughout the whole epithelia. Syncytia formed of ciliated and basal cells appeared at the apical side of the epithelia within 3 to 4 d and were released into the apical lumen, where they contributed to the transmittable virus dose. A small number of reconstituted epithelia were intrinsically more resistant to virus infection, limiting virus spread to different degrees. This phenotype was more frequent in epithelia derived from children versus adults and correlated with an accelerated release of type III interferon. Treatment of permissive adult epithelia with exogenous type III interferon restricted infection, while type III interferon gene knockout promoted infection. Furthermore, a transcript analysis revealed that the inflammatory response was specifically attenuated in children. Taken together, our findings suggest that apical syncytia formation is an underappreciated source of virus propagation for tissue or environmental dissemination, whereas a robust type III interferon response such as commonly seen in young donors restricted SARS-CoV-2 infection. Thus, the combination of interferon restriction and attenuated inflammatory response in children might explain the epidemiological observation of age-related susceptibility to COVID-19.

## Significance

Bronchial epithelia are the primary target for severe acute respiratory syndrome coronavirus 2 (SARS-CoV-2) infections. Here, we reconstituted bronchial epithelia from adult and child donors and show that SARS-CoV-2 infections spread fast, resulting in the formation and synchronized release of large clusters of infected cells and syncytia into the apical lumen, contributing to virus dissemination. Some epithelia, for the most part from children, revealed an intrinsic resistance to infection and virus spread. This infection control correlates with faster type III interferon secretion and can be transferred to permissive epithelia through exogenous interferon application. Child epithelia also showed a muted inflammatory response compared with adult, suggesting a specific and age-adapted epithelial response to SARS-CoV-2 infection that may explain why children are less susceptible to severe COVID-19.

In late 2019, clusters of patients with pneumonia were identified in Wuhan, China, and were subsequently shown to be infected with the novel severe acute respiratory syndrome coronavirus 2 (SARS-CoV-2) (1–3). SARS-CoV-2 infections are associated with acute respiratory illness referred to as COVID-19. Since its description, SARS-CoV-2 infections are at the root of an enduring worldwide pandemic, having caused over 5.4 million deaths and more than 300 million confirmed infections (https://coronavirus.jhu.edu/).

SARS-CoV-2 is an enveloped virus with a positive single-stranded RNA of around 30 kb. The virus particle contains four structural proteins, the genome packaging Nucleocapsid (N) and the transmembrane proteins Envelope (E), Membrane (M), and Spike (S) (4–6). The surface exposed Spike protein gives the virus its crown-like appearance in electron microscopy (EM) and mediates the attachment to the main cellular receptor ACE2 (7). Coronaviruses can cause a wide range of respiratory illnesses, from mild upper respiratory tract infections up to a severe acute respiratory syndrome (8). The latter is characterized by a delayed interferon (IFN) response, excessive cytological damage, and inflammation (9). Postmortem biopsies in patients that died from COVID-19 point to airways and lungs as primary targets of the disease (10, 11), with advanced diffuse alveolar damage, pulmonary thrombosis, and abnormal syncytia formation (12, 13). Several studies suggest that cytokine storm and inflammatory infiltrates in the alveolar space are associated with disease severity and death in response to COVID-19 (14, 15).

While SARS-CoV-2 is genetically close to SARS-CoV, it shows much higher effective transmissibility (16, 17). One reason for this higher contagiousness is an active virus replication in tissues of the upper respiratory tract at an early stage of infection, with large amounts of virus produced 4 d after the beginning of symptoms, and an active replication in the throat (18, 19). Furthermore, the viral load detected in asymptomatic patients was similar to that of symptomatic patients on the fourth day after symptom onset (20), suggesting equal transmission potential of asymptomatic or minimally symptomatic patients at very early stages of infection (21).

Epidemiological data show that all ages of the population are susceptible to SARS-CoV-2 infection; however, the SARS-CoV-2 infection severity differs between the child and adult population, consolidating a large discrepancy in death rates of SARS-CoV-2–infected patients associated with age (22). Children under the age of 9 y have very low fatality rates of SARS-CoV-2 infection (under 0.001%) compared to older patients (increasing to 8% in elderly patients >80 y). A recent metadata analysis of several studies confirmed this correlation between age and disease severity (23). Still, the reason for this age-related difference is not clear.

Only limited information is available considering the mechanisms of intraepithelial viral spreading and virus release, or interindividual transmission. Over the course of a 51-d period, infection of a reconstituted human airway epithelium infected with SARS-CoV-2 showed multiple waves of viral replication associated with the degradation of tight junctions and a decrease in the number of cilia in the epithelial cells (24, 25). In this model, plaque-like cytopathic structures could be observed with the formation of multinucleated cells reminiscent of fused cells forming syncytia (26). Syncytia formation due to Spike-ACE2 interactions were readily observed in cultured cells, but their pathophysiological role remains unclear (27, 28).

IFN induction appears to be limited in the most severe clinical cases (29–31). Viral RNA production in bronchial epithelia (BE) increased at 2 d postinfection (dpi). In contrast, release of IFN lambda (IFN λ) was induced at 4 dpi of BE, suggesting a delay in the induction of the cellular antiviral response. Recent reports studying cell-intrinsic changes occurring in cells derived from upper airways from children, adults, and the elderly infected with SARS-CoV-2 have shown that aging contributed to viral load, transcriptional responses, IFN signaling, and antiviral responses (32). Another study using single-cell transcript analysis from upper airway cells of different age group donors suggested that children mount an accelerated antiviral immunity in the upper respiratory tract due to elevated baseline levels of innate sensors such as MDA5 and RIG-I (33). Innate sensing of SARS-CoV-2 triggers type I and III IFN responses, and MDA5, and possibly RIG-I, are emerging as main specific pathogen recognition receptors (34–36). A strong correlation between severe COVID-19 and inborn errors in the IFN system further highlights the importance of the IFN response in SARS-CoV-2 infection control (31). Despite this emerging picture, studies addressing the SARS-CoV-2 infection process in the BE, a model mimicking the primary infection site, are limited. Here we reconstituted BE in air–liquid interface derived from BE samples of adult and child donors. We monitored the replication of SARS-CoV-2 over several days and followed virus spread in the epithelia. Imaging revealed the synchronized and vast formation and apical release of cells and syncytia occurring between 3 and 4 dpi. The released cells retained infectivity, suggesting they contribute to the spreading of the virus in the epithelium. Furthermore, we observed reconstituted BE, mostly from children, with very low viral production and restricted viral spread correlated to rapid type III IFN release. In addition, permissive child epithelia showed an attenuated inflammatory response compared to adults. These results may explain the clinical and epidemiological observations that SARS-CoV-2 infections have a more severe clinical manifestation in older patients.

## Results

### Generation of a Fully Differentiated BE Model.

Primary infections with SARS-CoV-2 often initiate in the upper respiratory tract, from which they can spread to the lower respiratory tract to cause severe disease (17). BE are pseudostratified cell layers with typical tight junctions to connect the epithelia layer, as well as a mucus layer and beating cilia on the lumen side (37, 38). To study the SARS-CoV-2 infection process in a physiologically relevant model, we established a cellular in vitro model of BE differentiated in air–liquid interface from individual donors (*SI Appendix*, Fig. S1). Primary BE cells were collected from surgical bronchial resection or fibroscopy from individual adult donors collected in the bronchial tree between the third and fifth generation at Bordeaux University hospital. Patients were between 29 and 74 y old, with a normal body mass index (BMI) (Table 1). Moreover, all donors had normal or subnormal lung function, with a percentage of FEV1 ≥ 70 and a FEV1/FVC ratio ≥ 0.7. Basal epithelial cells were expanded in vitro and differentiated on cell culture inserts at the air–liquid interface for ∼21 d (*SI Appendix*, Fig. S1*A*). This differentiation protocol generated between 12 and 24 individual inserts from a single donor, allowing comparative analysis. Immunofluorescence (IF) analysis confirmed the presence of differentiated cell types. Detection of acetylated tubulin (AcTub), mucin 5A (Muc5A), and cytokeratin 5 (CytK5) identified multiciliated cells, goblet cells, and basal cells, respectively (*SI Appendix*, Fig. S1 *B* and *C* and Movies S1 and S2), confirming the pseudostratified apical-to-basolateral organizational integrity of the BE, for example, a single-cell layer of apical multiciliated cells covering a layer of basal cells. This organization was verified by EM, showing the presence of well-differentiated cilia and tight junctions (*SI Appendix*, Fig. S1*D*). Next, we determined the localization of ACE2, the primary receptor for SARS-CoV-2, using IF analysis (*SI Appendix*, Fig. S1*E* and Movie S3). Colabeling with antibodies against ACE2 and AcTub confirmed that ACE2 was expressed in apical multiciliated cells as previously reported (2, 39). Moreover, our data showed the pronounced exposure of ACE2 on individual cilia reaching into the apical lumen, which suggests that the ACE2 entry receptor can be easily accessed by viruses from the respiratory tract.

**Table 1.** Clinical and functional characteristics of subjects

Characteristic	Adults	Children
No. of patients	12	7
Sex (M/F)	5/7	3/4
Age, y	58.67 ± 2.46	9.02 ± 1.36
Range, y	29–74	4–12
Body mass index, kg/m^2^	23.20 ± 0.72	15.51 ± 0.72
Lung function		
Smoking status (no. current/former/ nonsmoker)	2/7/3	NA
FEV1%	92.20 ± 7.74	86.20 ± 19.1
FEV1/FVC ratio	0.83 ± 0.01	0.81 ± 0.05

Data are presented as mean ± SEM. Lung function was measured only in four children, and was not measured in the three children under 5 y old. FEV_1_, forced expiratory volume in 1 s, FVC, forced volume capacity; NA, not applicable.

### SARS-CoV-2 Monitoring and BE Infection.

**Fig. 1.**
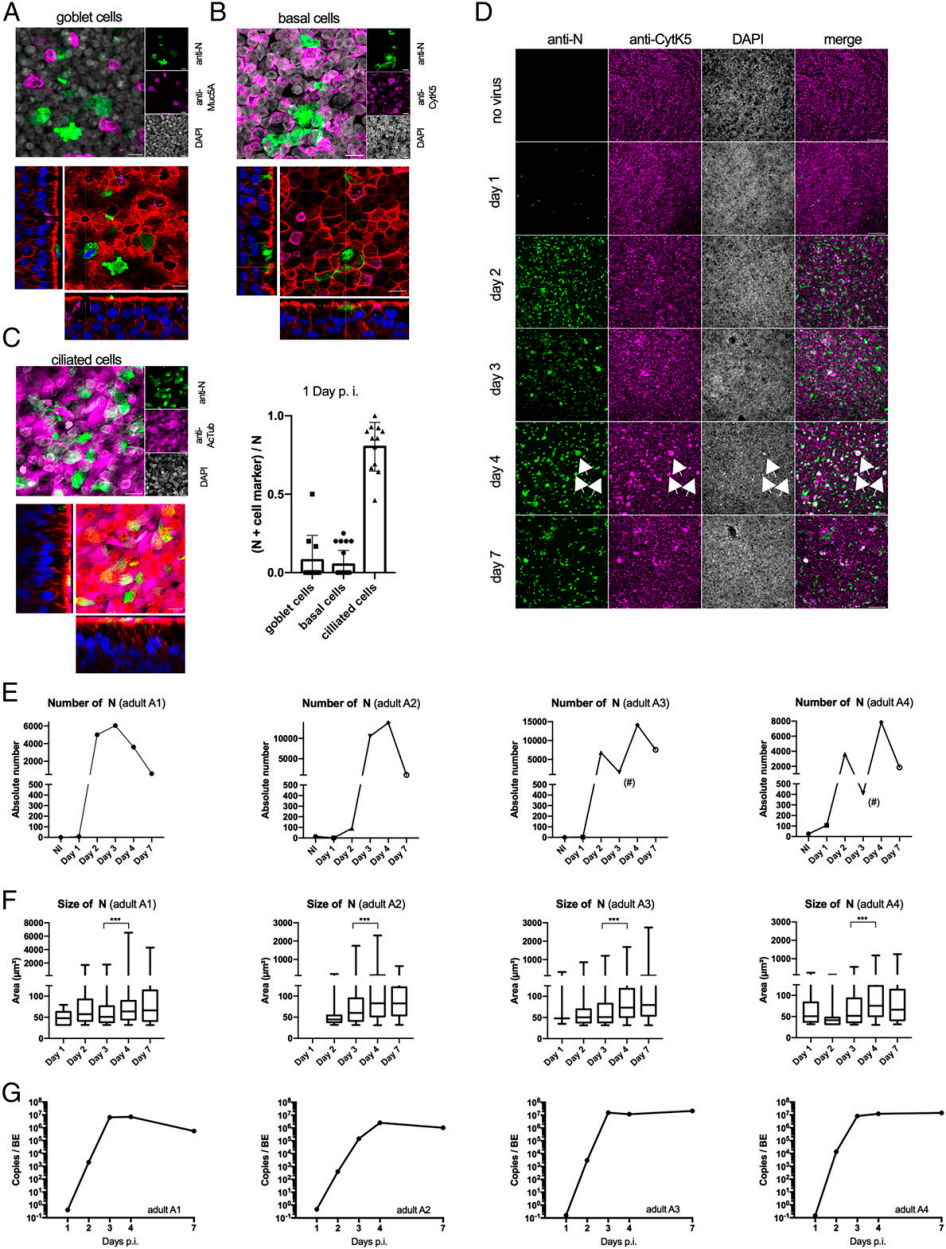
SARS-CoV-2 infection of BE. (*A*) Differentiated BE were infected with SARS-CoV-2 and stained 24 h postinfection with anti-N (green signal) to identify infected cells and with anti-Muc5A to detect goblet cells (magenta signal) and counterstained with DAPI (gray signal). *Top* shows a Z projection, and *Bottom* shows an individual Z section counterstained with phalloidin (red signal) and DAPI (blue signal). (Scale bar, 10 µm.) For full Z stack, see Movie S4. (*B*) As in *A* but stained with anti-N (green signal) and anti-CytK5 to identify basal cells (magenta signal). (Scale bar, 10 µm.) For full Z stack, see Movie S5. (*C*) (*Left*) As in *A* but stained with anti-N (green signal) and anti-AcTub to identify multiciliated cells (magenta signal). (Scale bar, 10 µm.) For full Z stack, see Movie S6. (*Right*) Quantification of the colocalization of N vs. AcTub, CytK5 or Muc5A for at least 11 images per marker from one or two donors. (*D*) SARS-CoV-2 infection kinetic of BE: representative widefield microscopy images of BE from adult donor (A3) at low resolution. (Scale bar, 50 µm.) BE were fixed at days 1, 2, 3, 4, and 7 as indicated to the left of each row; noninfected controls were also fixed at day 7. BE were stained with anti-N antibodies to detect infected cells (green signal, first column) and anti-CytK5 to detect basal cells (magenta signal, second column), counterstained with DAPI (gray signal, third column), and merged (fourth column). Large specific signals in all channels are apparent on day 4 (white arrows). (*E*) The absolute number of N-positive signals was determined for each BE for the whole epithelia on each day and is shown as absolute number of N dots at different dpi (detailed in *SI Appendix*, *Supplemental Methods (Imaging)*); ^#^partial BE damage. (*F*) Signals quantified in *E* were classed by size and plotted as min to max box and whisker plots, ***P < 0.001 based on one-way ANOVA analysis. (*G*) Apical washes for each BE were subjected to RT-qPCR analysis to determine genome copy numbers at days 1, 2, 3, 4, and 7 postinfection as indicated.

Next, BE were inoculated on the apical side with 1,200 plaque-forming units (PFU) of SARS-CoV-2 for 1 h, followed by inoculum removal. Apical and basolateral compartments were collected 3 dpi and used to infect Vero E6 cells for 3 d. Infectivity was determined 3 dpi by endpoint titration, showing a median tissue culture infectious dose (TCID_50_) between 5.24 × 10^4^/mL and 1.68 × 10^4^/mL (corresponding to 36,156 and 11,592 PFU/mL) for the apical compartment. Infectivity for the basolateral compartment was below the detection limit (20.7 PFU/mL). These data demonstrated that SARS-CoV-2 actively replicates in reconstituted BE and that newly produced virions are released from the apical side 3 dpi. To detect virus-infected cells, we generated monoclonal antibodies against the SARS-CoV-2 nucleocapsid protein (N). Clone 3G9 was selected, which specifically recognized N (*SI Appendix*, Fig. S2*A*) and stained infected cells (*SI Appendix*, Fig. S2*B*). To identify the primary cell target during SARS-CoV-2 infection, fully differentiated BE were infected following the above infection protocol. BE were fixed 1 dpi and processed for IF analysis using SARS-CoV-2 N-specific antibodies (Fig. 1 *A*–*C*). Specific colabeling with antibodies against Muc5A (Fig. 1*A* and Movie S4) or CytK5 (Fig. 1*B* and Movie S5) showed that neither goblet nor basal cells were the primary cell target at the beginning of the infection. Conversely, at 1 dpi, anti-N staining was systematically associated with staining for AcTub, a specific marker for multiciliated cells (Fig. 1*C* and Movie S6). Quantification revealed that over 80% of infected cells costain with the ciliated cell marker, confirming that ciliated cells are the primary target (Fig. 1 *C*, *Right*). This is consistent with previous reports that apical multiciliated cells are the primary target cells for SARS-CoV-2 infection (2, 26, 40). BE were also costained with fluorescent phalloidin to mark cell boundaries for three-dimensional (3D) imaging of the entire epithelial depth (3D imaging; displayed as Movies S1–S12). Infected cells were exclusively located at the apical surface of the BE, confirming TCID_50_ results in the apical vs. basolateral compartment.

To understand how SARS-CoV-2 spreads in the epithelium after initial infection of multiciliated cells, we next infected BE from several individual adult donors and monitored them over the course of 7 d. For four adult donors (A1 to A4), we used IF detection of infected cells and performed image analysis at different dpi (Fig. 1). Anti-N-specific signals could be detected as soon as 1 dpi in a small number of cells. The number of infected cells and signal intensity increased drastically starting at 2 dpi and decreased slightly toward the end of the 7-d observation period (Fig. 1*D*, adult A3). Similar results were obtained with the other adults, suggesting rapid onset of viral replication and spread (*SI Appendix*, Fig. S3). We next quantified the number of anti-N-positive signals for each donor, confirming that the number of infected cells strongly increased within 2 dpi to 3 dpi, reaching a maximum around 4 dpi, and decreased somewhat by 7 dpi (Fig. 1*E*). At the peak of infection, the staining with anti-N antibodies revealed several enlarged structures. These structures colabeled with CytK5, the marker for basal cells (see arrows in Fig. 1*D* and *SI Appendix*, Fig. S3), and appeared at 3 dpi but were most prominent at 4 dpi for all donors. This was confirmed by quantifying the size of the anti-N signals over time (Fig. 1*F*), showing a statistically significant increase in average signal size between 3 and 4 dpi. In parallel, release of newly produced viruses into the apical lumen was quantified by RT-qPCR (Fig. 1*G*). For all four donors, the viral RNA copy number present in the lumen correlated with the observed cellular anti-N labeling, with a fast increase from 2 dpi reaching a plateau between 3 and 4 dpi. These data confirm that apical SARS-CoV-2 inoculation of BE resulted in efficient infection and subsequent progeny production and release into the apical lumen.

### Infected Multiciliated Cells Form Syncytia with Basal Cells at the Apical Side of the BE.

**Fig. 2.**
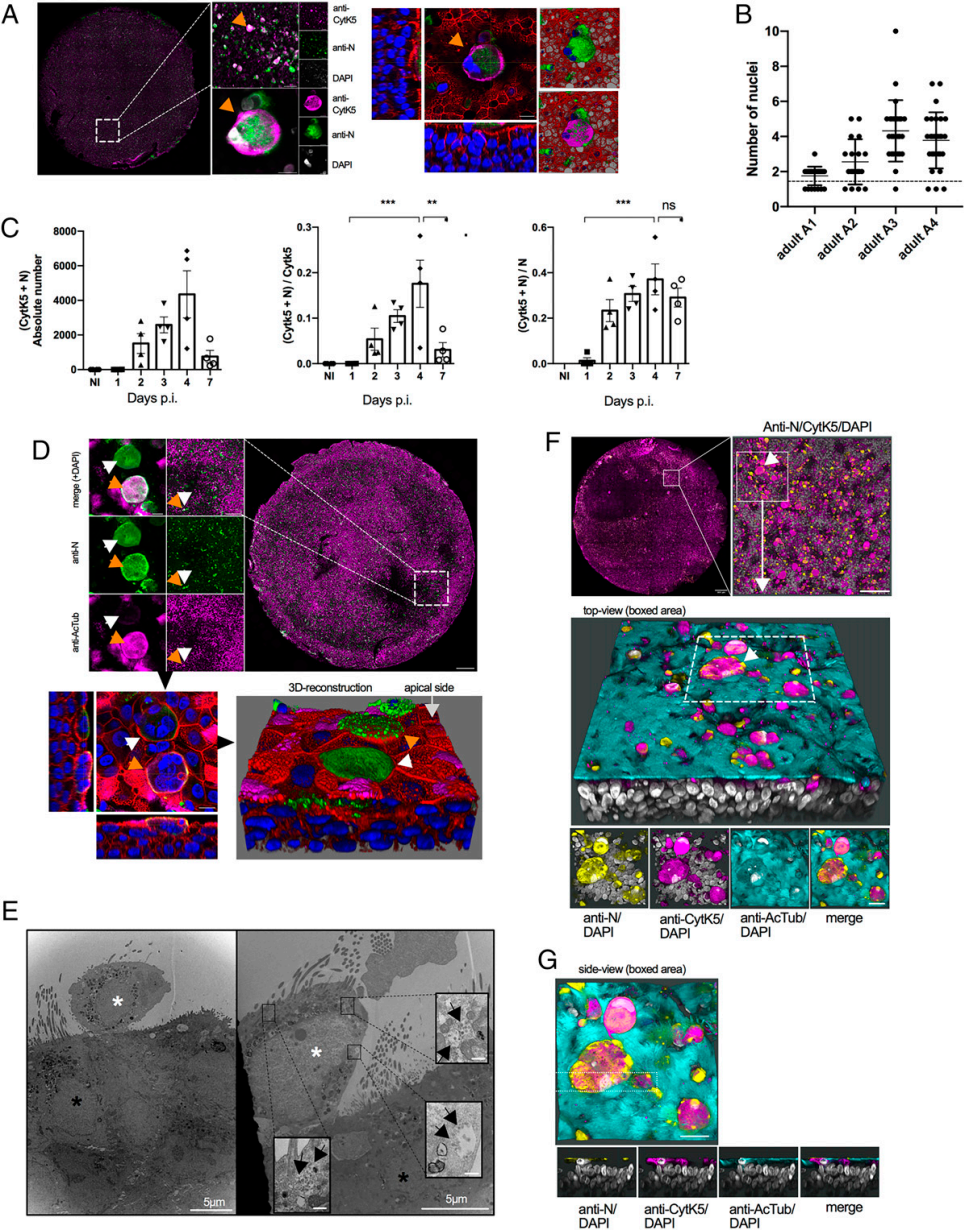
SARS-CoV-2 infection of BE produces apical syncytia. (*A*) Analysis of entire BE 4 dpi (*Left*). BE were stained with anti-N (green signal) and anti-CytK5 antibodies (magenta signal) and counterstained with DAPI (gray or blue signal). Individual syncytia (orange arrow) are further magnified as maximum Z projection (*Left*) or as individual Z stack (*Right*, with phalloidin counterstain in red), or as 3D image reconstruction to see its apical location. (Scale bar, 10 µm for the right panel and magnified bottom left panel, 50 µm for the magnified top left panel and 500 µm for the BE overview) See also Movie S7. (*B*) Quantification of the number of nuclei per syncytia-like structure for donors A1 to A4 (*n* = 30 per epithelia). Multinucleated cells are above the dashed line. (*C*) Quantification of the total number of double-positive cells (N and CytK5, *Left*) and normalized for total number of basal cells (*Middle*) and total number of infected cells (*Right*) using semiautomatic quantification. Data are presented as mean ± SD, *n* = 4 (donors A1 to A4). ***P < 0.001, *P < 0.05, ns = non significant, based on one-way ANOVA analysis. (*D*) Analysis of entire BE 4 dpi as in *A*. BE were stained with anti-N (green signal) and anti-AcTub antibodies (magenta signal) and counterstained with DAPI (upper panel, gray or lower panel, blue signal) and represented as 3D image reconstruction with phalloidin counterstain (lower panel, red signal). Double-positive syncytia are marked by an orange arrow, and single-positive syncytia are marked by a white arrow. (Scale bar, 10 µm for the highest magnification (upper left and 3D image), 200 µm for the intermediate magnification (upper middle), and 500 µm for the BE overview.) See also Movie S8. (*E*) Electron micrograph of infected BE 4 dpi. The large images show extruded cell on the apical side of the epithelia (white asterisk) adjacent to multiciliated cells (black asterisk). *Insets* show virus-containing vacuoles in the extruded cell indicated by black arrows. (Scale bars, 5 µm for large images and 200 nm for *Inserts*). (*F*) Analysis of entire BE 4 dpi as in *A*. BE were triple labeled and pseudo colored following staining with anti-N (yellow signal), anti-CytK5 (magenta signal), and anti-AcTub antibodies (cyan signal) and counterstained with DAPI (gray signal). A syncytia (marked by white arrow)-containing area is shown as a lateral 3D reconstruction. The boxed area is further shown as top view with individual signal combinations as indicated. (Scale bar, 20 µm for the mag regions and 500 µm for the BE overview.) See also Movie S9 a–c. (*G*) Image and analysis as in *F* with area shown as side view, cut through the syncytia (boxed area) with individual signal combinations below as indicated.

The enlarged N-positive structures forming at 3 dpi to 4 dpi were revealed as multinucleated cells reminiscent of syncytia when observed at high magnification. They could be found in all areas of the infected epithelia derived from the four donors but were absent in noninfected control BE (Fig. 1 and *SI Appendix*, Fig. S3). Unexpectedly, the N-positive syncytia-like structures also stained positive for the basal cell marker CytK5 (Fig. 2*A*), despite the fact that basal cells were rarely infected and did not form syncytia-like structures at earlier time points. To verify that these structures were indeed multinucleated syncytia, we quantified the number of nuclei from ∼30 syncytia-like structures, randomly at 4 dpi, selected based on the N signal for each of the four donors (Fig. 2*B*). Several analyzed structures had multiple nuclei, with two of the donors (A3 and A4) having a larger average number of nuclei and thus likely being in an advanced infection state. This analysis confirmed that syncytia are formed upon BE infection within 3 dpi to 4 dpi. To better understand syncytia formation, we next quantified the number of double-positive cells (i.e., anti-N and anti-CytK5) over time (Fig. 2*C*). The proportion of double-positive cells (i.e., syncytia) increased constantly and reached a maximum at 4 dpi, after which we observed a drastic drop in the number of double-positive cells (Fig. 2 *C*, *Left*). Normalization of the double-positive cells for either the total number of basal cells (Fig. 2 *C*, *Middle*) or the total number of infected cells (Fig. 2 *C*, *Right*) revealed that double-positive cells, but not total infected cells, disappeared at 4 dpi. When observed at high magnification, we saw that syncytia in different regions of the BE also frequently lost their stain for AcTub (Fig. 2 *D*, *Upper*). Syncytia that still expressed AcTub showed an amorphous staining pattern, rarely distinguishing cilia features. Part of the syncytial structures also failed to stain with phalloidin used to delineate the cellular actin cortex (e.g., Fig. 2 *D*, *Lower*). Furthermore, 3D imaging showed that syncytia formed exclusively on the apical side of the BE, often identified as an elevated layer on top of the epithelia (Fig. 2 *A*, *Right* and Fig. 2 *D*, *Bottom*; see also Movies S7 and S8). Such cell extrusions in infected epithelia were also observed using EM (Fig. 2*E*, white asterisk), but never in the context of noninfected epithelia (*SI Appendix*, Fig. S1). Extruded cells had only reminiscent cilia structures, unlike multiciliated cells in noninfected epithelia, consistent with a previous report that SARS-CoV-2 infection of lung epithelial cells triggers partial loss of cilia (25). Importantly, using EM, we observed vesicular inclusions within the extruded cells that contained viral particles, indicating that multinucleated infected cells actively produced viruses (Fig. 2*E*, black arrows). Taken together, our IF analysis strongly suggested that syncytia were formed through the fusion of infected ciliated cells with basal cells, coinciding with the loss of cilia and reorganization of cytoskeletal features including the actin and tubulin cytoskeleton. To validate this assumption, we performed triple labeling at 4 dpi with antibodies against basal and ciliated cell marker (Cytk5 and AcTub) and identified infected cells via N stain (Fig. 2*F*). This analysis confirmed that syncytia formation occurred only on the apical side of the BE, commonly showing loss of cell identity defining features. Moreover, syncytia stained positive for both cell markers (i.e., Cytk5 and AcTub), indicating a possible fusion event between ciliated and basal cells (top view in Fig. 2*F* and side view in Fig. 2*G*; see also Movie S9).

### Infected Cells and Syncytia Are Released into the Apical BE Lumen and Transmit Infection.

**Fig. 3.**
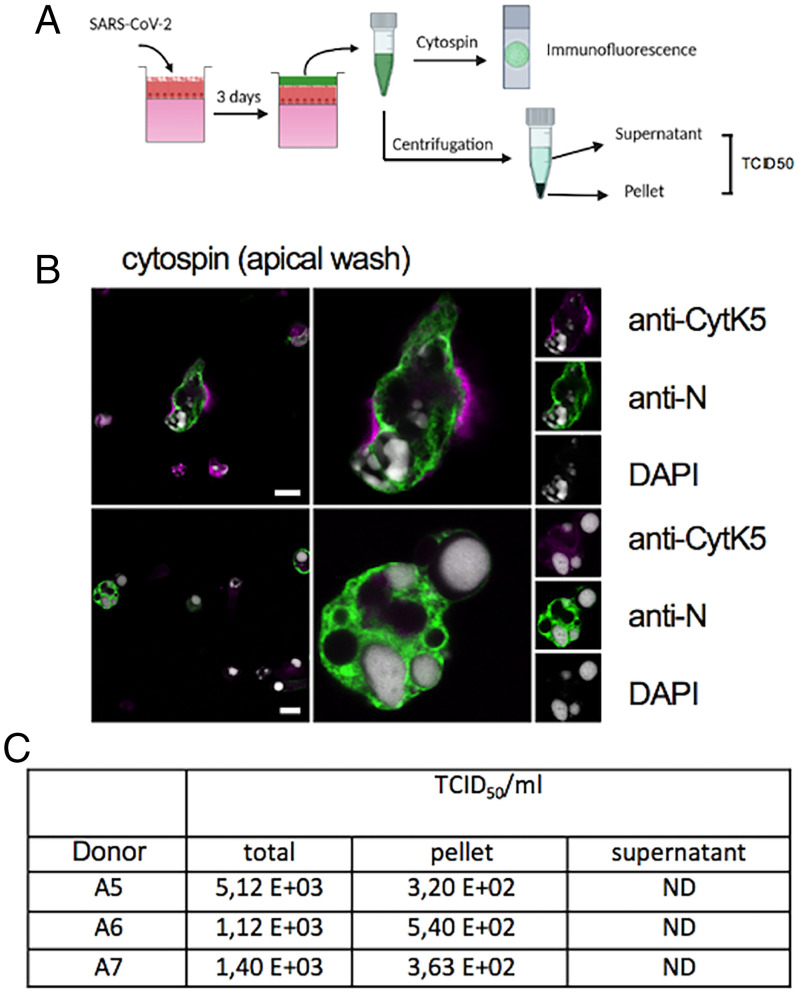
Apical released cells and syncytia transmit infection. (*A*) Experimental design. Apical washes from three donors (A5 to A7) were fixed at 3 dpi and concentrated on slides using cytospin (*Top*) or collected and separated into supernatant and cell pellet and used to infect Vero E6 cells (*Bottom*). (*B*) Representative image from cytospin, stained with anti-N (green signal) and anti-CytK5 antibodies (magenta signal) and counterstained with DAPI (gray signal). Arrows indicate syncytia in the overview and magnified to the left. Individual channels are as indicated. (Scale bar, 10 µm.) (*C*) Infectivity determination of apical washes for three donors determined by TCID_50._ ND, nondetected.

Because cells and syncytia were extruding from the epithelium, we wondered whether infected cells/syncytia could be released from the epithelium and account for the spreading of the infection. To test this hypothesis, we infected epithelia from three donors. At 4 dpi, apical BE washes were concentrated on microscope slides via cytospin for IF analysis or used for infectivity studies (Fig. 3*A*). The IF analysis showed that apical washes contained individual infected cells but also several virus-containing syncytia, showing their accumulation in the apical BE lumen (Fig. 3*B*). To test the relative infectivity, apical washes were separated by centrifugation into a cell-containing fraction and a cell-free supernatant (Fig. 3*C*, pellet and supernatant). The cellular fraction was resuspended in an equal volume of fresh medium, and both fractions were subsequently used to infect Vero E6 cells and compared to total apical washes infectivity (total). The relative infectivity of both fractions was determined 3 d later by endpoint titration. Surprisingly, only the fraction containing cells/syncytia showed significant infectivity after 3 d, while the cell-free supernatant had not resulted in measurable infectivity at this time (Fig. 3*C*). Taken together, this analysis suggests that BE produce and release virus into the apical lumen. Importantly, a significant fraction of the infectious virus dose stems from cell-associated virus, including syncytia, although entrapment in mucus cannot be excluded.

### BE from Children Exhibit Differential Levels of SARS-CoV-2 Infection.

**Fig. 4.**
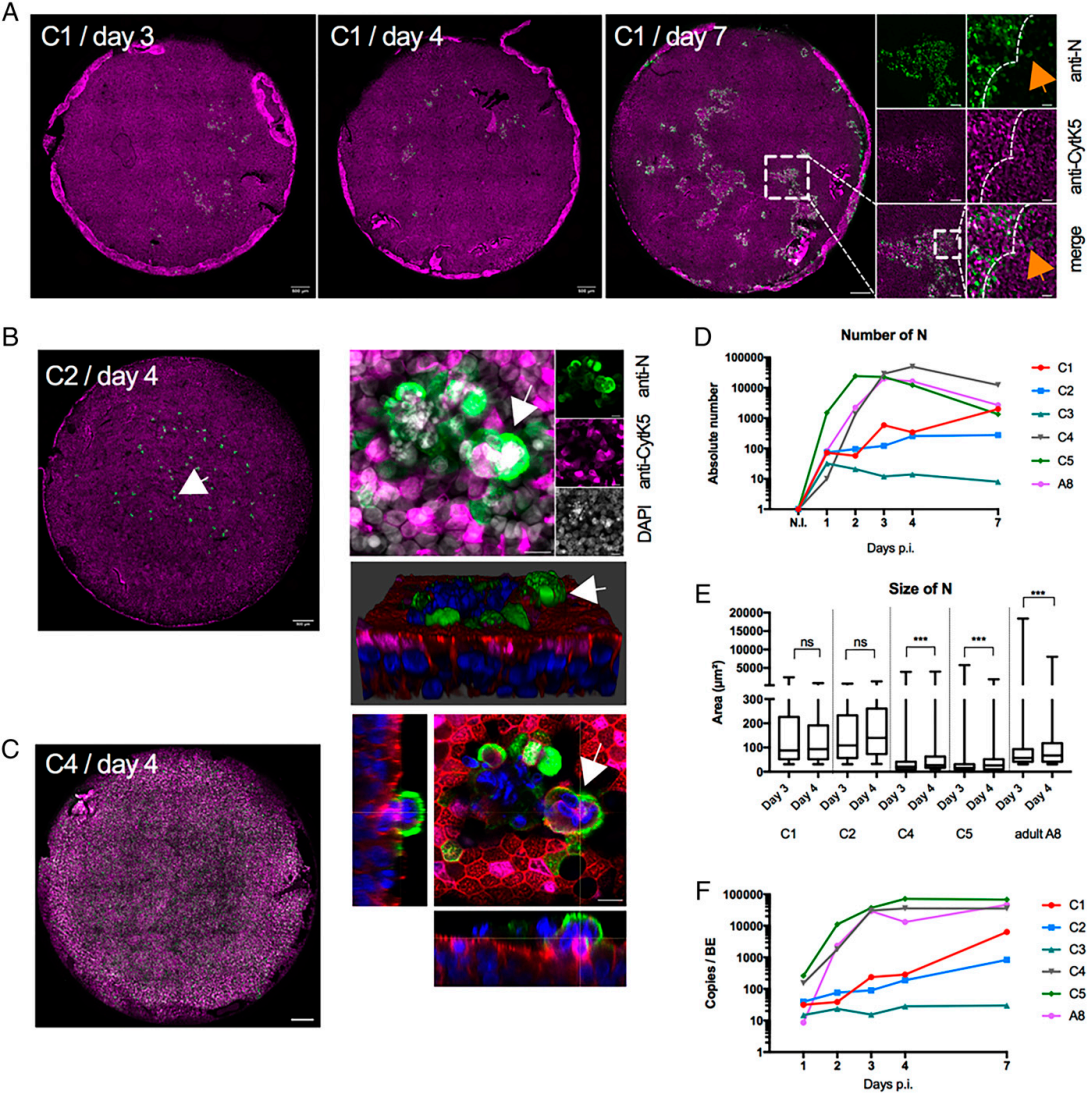
SARS-CoV-2 infection of BE from children. (*A*) BE Infection kinetic from donor C1 (C, child). Entire BE are shown, and dpi is indicated on the top. BE were stained with anti-N antibodies to detect infected cells (green signal) and anti-CytK5 marking basal cells (magenta signal). The boxed area containing infection foci is magnified to the right. The higher magnification shows the infection front (dashed line) and individual infected cells in the vicinity of the infection front (orange arrow). (Scale bar, 20 µm for the highest magnification of the boxed area, 100 µm for the intermediate magnification of the boxed are and 500 µm for the BE overview) (*B*) (*Left*) BE from donor C2 at 4 dpi presented as in *A*. The white arrow in the entire BE points at a syncytium further magnified as a maximum Z projection (*Right Top*) or individual Z stack (*Right Bottom*, with phalloidin counterstain in red), or as a 3D image reconstruction to see its apical location. (Scale bar, 10 µm for the agnified areas and 500 µm for the BE overview.) See also Movie S12. (*C*) Entire BE of permissive donor C4 at 4 dpi. Staining is as in *A*. (Scale bar, 500 µm.) (See *SI Appendix*, Fig. S4 for further examples.) (*D*) Quantification of N-positive cells for the entire BE for three permissive (C1 to C3) and two nonpermissive (C4 and C5) children and one permissive adult (A8). Color code is as indicated. (*E*) The average size of N-positive signals from *D* at 3 and 4 dpi classed by size and plotted as min to max box and whisker plots. Statistical analysis was done on the whole time course for each donor (as in *D*); only days 3 and 4 postinfection are shown. ***P < 0.001 based on one-way ANOVA as indicated. (*F*) Apical washes for permissive and nonpermissive BE were subjected to RT-qPCR analysis to determine genome copy numbers at days 1, 2, 3, 4, and 7 after infection. Color code as indicated and same as in *D*.

**Fig. 5.**
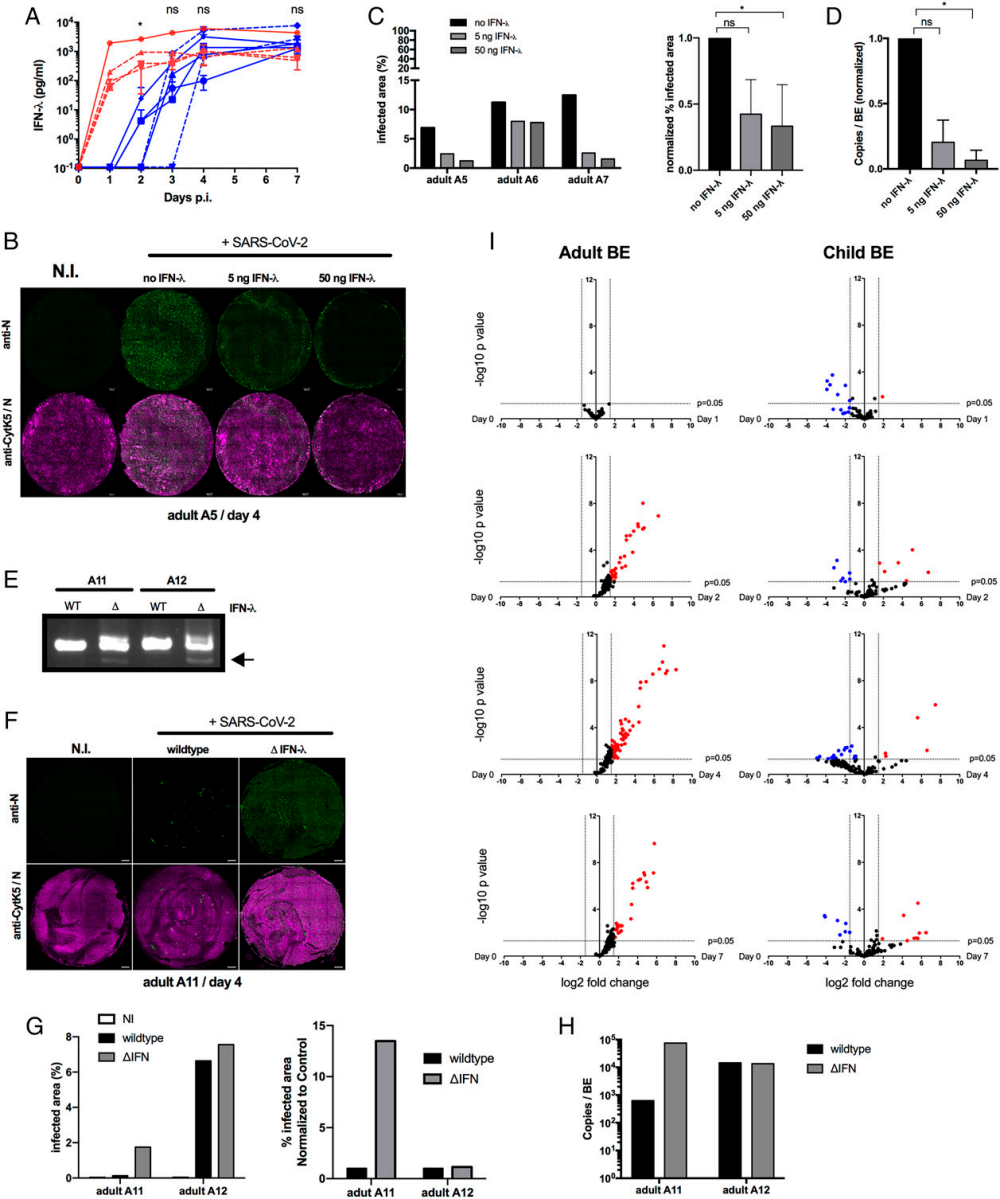
Secretion of IFN-λ from BE. (*A*) Basolateral supernatant from permissive (blue) and nonpermissive (red), corresponding to children (dashed line, *n* = 5) and adult (solid lines, *n* = 5) donor BEs were subjected to ELISA to determine IFN-λ concentration at days 0, 1, 2, 3, 4, and 7 postinfection as indicated. Results are presented as mean ± SEM. *P < 0.05, unpaired *t* test per day permissive vs. nonpermissive. (*B*) BE Infection following IFN-λ treatment. Entire BE are shown at 4 dpi stained with anti-N antibodies to detect infected cells (green signal) and anti-CytK5 marking basal cells (magenta signal). (Scale bar, 500 µm.) (*C*) Quantification of infected area for three different donors (A5 to A7) following IFN-λ treatment as indicated. Data are shown as percentage of total infected BE area (*Left*) and normalized to nontreated control (*Right*). *P < 0.05, based on one-way ANOVA analysis. (*D*) Apical washes of each donor following IFN-λ treatment were subjected to RT-qPCR analysis to determine genome copy numbers. Data are shown normalized to nontreated control. *P < 0.05, based on one-way ANOVA analysis. (*E*) Verification of CRISPR/Cas9 gene knockout for donors A11 and A12. PCR amplification is of a region encompassing the deletion in IFNλ2 gene. Gene deletion–specific PCR product is indicated by the arrow. (*F*) BE Infection following IFN-λ gene deletion. Entire BE from donor A11 are shown at 4 dpi stained as in *B*. (*G*) Quantification of infected area for two different donors (A11 and A12) following IFN-λ gene deletion and WT donor control as indicated. Data are shown as percentage of total infected BE area (*Left*) and normalized to nontreated control (*Right*). (*H*) Apical washes of donors A11 and A12 at 4 dpi were subjected to RT-qPCR analysis to determine genome copy numbers. (*I*) Transcript analysis of adult (*Left*, *n* = 4) and child (*Right*, *n* = 4) BE using a panel of inflammation response genes (see *SI Appendix* for details, and see Dataset S1 for gene list). For adults and children, transcript expression was compared between noninfected control BE and 1, 2, 4 and 7 dpi. The data show gene expression changes for adults (*Left*) and children (*Right*) for days 1, 2, 4, and 7 (*Top* to *Bottom*). Volcano plots show fold change compared to noninfected control as indicated as log2. Significant transcriptional activation (red) or transcriptional repression (blue) was defined for target genes with a fold expression >1.5 log2 or <−1.5 log2 (corresponding to a 2.5-fold increase or decrease, respectively) and −log10 *P* values > 1.35 (corresponding to *P* values < 0.05) as indicated (gene ID and measured induction levels are provided in Dataset S2).

Each BE sample can be traced to an individual donor. While almost all of the adult donors were highly permissive to SARS-CoV-2 infection, we observed a more restricted pattern of infection for a rare number of adult donors with low virus production in the apical lumen at 3 and 4 dpi quantified by RT-qPCR (see adult A11 in Fig. 5). The adult donors in this study were between 29 and 74 y old, with a mean of 58 y old (Table 1), which places them, statistically, into either a medium- or high-risk group to develop severe COVID-19. Children, on the other hand, are a group much less susceptible to severe forms of COVID-19 (41, 42). To investigate how SARS-CoV-2 infects child BEs, we next prepared epithelia from BE cells obtained from children aged 5 y to 12 y that have undergone bronchial fibroscopy (Table 1). Fully differentiated BE from children showed the same cellular arrangement (epithelial cells, basal cells, goblet cells) and physiological properties (cilia beating, mucus production) as adult epithelia. To characterize the SARS-CoV-2 infection dynamics, child BE and two independent adult controls were infected, and virus production was monitored by RT-qPCR. In addition, BE were fixed at 1, 2, 3, 4, and 7 dpi, including noninfected donor controls. Infection of the adult BE resulted, again, in rapid infection and progeny production. In contrast, the child BE showed distinct infection kinetics. When the BE were processed for IF analysis using antibodies against CytK5 and N, three out of five child BE (C1 to C3; *SI Appendix*, Fig. S4) showed a severely restricted infection profile, while the two others (C4 and C5; *SI Appendix*, Fig. S4) showed an infection kinetic comparable to the adult BE (e.g., A8; *SI Appendix*, Fig. S4). Interestingly, each restricted child BE had unique features. In BE derived from donor C1, we observed a slow but substantial increase in infected cells over time (Fig. 4*A*). Consecutive IF analysis of the entire epithelia from donor C1 revealed that initial infections were limited to a few cells that grew over time into infection foci and further enlarged by infecting surrounding cells at their periphery. Cells at the foci border stained strongly, while several cells in the foci vicinity stained weakly positive for SARS-CoV-2 N. This suggested a front of highly replicating cells with forward cell-to-cell or short-range spread as the infection mode (Fig. 4 *A*, * magnified boxed area*). In comparison, BE derived from donor C2 showed a much slower increase in the number of infected cells after the initial appearance of positive cells and strongly restricted infection spread (Fig. 4 *B*, *Left*). For C2, most of the initially infected cells developed into local clusters of infected cells without much lateral spread. Some clusters contained infected cells fused to form syncytia with basal cells with apical localization (Fig. 4 *B*, *Right* and Movie S10), reminiscent of syncytia formation in adult BE. Lateral spread into small foci could only be observed at 7 dpi, resembling observations made for donor C1 at earlier time points (2 dpi to 3 dpi). The BE derived from C3 only ever showed very few infected cells throughout the whole observation period, suggesting an abortive infection (*SI Appendix*, Fig. S4 *D* and *G*, *Left*). In contrast, BE from the permissive child C4 (Fig. 4*C*) and C5 (*SI Appendix*, Fig. S4 *F* and *G*, *Middle*) or adult donor controls (*SI Appendix*, Fig. S4 *A* and *G*, *Right*) showed fast increase in cell infection (within 2 d) and extensive formation of syncytia at 4 dpi, as previously observed. Quantifying the number of infected cells or cell cluster between nonpermissive child BE vs. permissive child BE and the adult control confirmed the slower infection progress (Fig. 4*D*). This included a more heterogeneous signal size distribution for infected cells, with no significant differences observed between day 3 and day 4, unlike for the permissive children or the adult (Fig. 4*E*). By comparison, IF analysis of the permissive child BE showed no difference from the adult BE, neither in the overall number of infected cells nor in the size distribution of syncytia formation or the kinetics of their appearance (see *SI Appendix*, Fig. S4 for representative experiment). The different staining profile corresponded to the quantification of viral replication determined by RT-qPCR (Fig. 4*F*). Taken together, our analysis of SARS-CoV-2 infection and spread in child BE demonstrated the ability of some child BE to restrict SARS-CoV-2 infection, while others remained permissive. This ability was not limited to child BE, as it was found in a very few adult BE (see adult A11 in Fig. 5). Consequently, we did not observe significant differences between children and adult BE in terms of size and number of infected cells when we compared permissive and nonpermissive BE jointly (*SI Appendix*, Fig. S4*H*), suggesting that it is not only the age but also the initial susceptibility to SARS-CoV-2 infection that determines BE infection spreadingadult A11 in figure 5.

### An Accelerated Type III IFN Response Protects BE from SARS-CoV-2 Infection.

Our observations suggested that not only child but, in rare cases, also adult BE could present intrinsic factors restricting SARS-CoV-2 infection. Possible explanations could be differences in the IFN response (43, 44), or morphological differences (45). The importance of type III IFN for SARS-CoV-2 control was recently shown (34). Accordingly, we compared the accumulation of IFN-λ and measured its concentration in BE medium from adults and children in response to SARS-CoV-2 infection (Fig. 5*A*). IFN λ was not detected for any of the BE at the beginning of the infection. However, child BE with restricted infection phenotype (C1 to C3) secreted IFN-λ starting at 1 dpi, whereas permissive BE from children and adults produced a detectable amount of IFN-λ only after 3 and 2 dpi, respectively. The only analyzed nonpermissive adult BE (A10) also produced IFN-λ at 1 dpi, similar to the corresponding nonpermissive child BE. IFN λ concentration increased subsequently for all groups and reached a plateau at 4 dpi to 7 dpi (Fig. 5*A*). The difference in the kinetics for IFN-λ secretion in response to SARS-CoV-2 infection could thus provide an explanation of why we observe BE that better resist SARS-CoV-2 infection.

To confirm the role of IFN-λ in SARS-CoV-2 spread in our model, we next investigated infected BE from three adult donors (A5 to A7) in the presence of increasing amounts of recombinant IFN-λ to mimic an accelerated IFN response. At 4 dpi, whole BE were stained for virus spread with and without IFN treatment for all donors (Fig. 5*B* and *SI Appendix*, Fig. S5*A*). We quantified the infection spread as percentage of the BE surface staining positive for N and revealed a strong dose-dependent inhibition of virus spread for two of the three donors, while the third donor showed only a slight reduction in virus spread (Fig. 5*C*). The protective effect of the IFN-λ was confirmed by RT-qPCR quantification of the viral RNA produced in the apical lumen (Fig. 5*D*). Interestingly, while IFN-λ reduced the total number of infected cells, it did not prevent syncytia formation per se, because the ratio of CytK5 positive infected cells (indicative of syncytia formation) was similar between IFN-λ treated and nontreated control BE (*SI Appendix*, Fig. S5*B*). Still, very few actual syncytia-resembling structures were observed at 4 dpi in IFN-λ treated BE with reduced infection, which probably reflects a less advanced infection state (*SI Appendix*, Fig. S5*B*).

To independently assess the role of IFN-λ in SARS-CoV-2 infection of BE, we analyzed the infection of BE from two adults (A11 and A12) following IFN-λ gene knockout using CRISPR/Cas9 applied to half the epithelia generated from each donor, while keeping unedited BE as a control. Partial gene KO was verified by PCR analysis (Fig. 5*E*). Interestingly, infection of the nonedited controls revealed that adult A11 was one of the few nonpermissive donors with very low infection rates. Adult A12, in contrast, was permissive, with high infection rates. In comparison, the edited ΔIFN-λ BE from donor A11 showed a strong increase of SARS-CoV-2–infected cells and progeny production compared to its nondeleted WT control (A11; Fig. 5*F*), whereas ΔIFN-λ deletion did not affect virus spread in donor A12 (A12 in Fig. 5*G* and *SI Appendix*, Fig. S5*C*). Quantification by RT-qPCR in the apical lumen confirmed the data of virus release (Fig. 5*H*). The observed difference between both donors suggests that IFN-λ control of SARS-CoV-2 infection is most efficient at low initial infection rates.

Our BE model revealed a protective role for IFN-λ. Severe cases of COVID-19 following SARS-CoV-2 infections are often linked to strong cytokine induction triggering an excessive inflammatory response (9). To further understand the response of child and adult BE, we performed a transcriptomic analysis using the nanostring technology (see *SI Appendix* for details) at 1, 2, 4, and 7 dpi, applying a panel of 255 genes involved in the inflammatory response. Only permissive samples were available for the experiment. Noninfected controls of adult (*n* = 4, A1 to A4) and child (*n* = 4, C4 to C7) BE revealed obvious age-dependent differences in the inflammatory response (Fig. 5*I*). Adult BE showed a strong transcriptional activation of proinflammatory genes starting at 2 dpi, which was maintained even after 7 dpi. In contrast, the response in child BE was much more attenuated compared to the adult BE (Fig. 5*I*), with very few up-regulated genes throughout the time course. Remarkably, the difference in inflammatory response did not affect virus spread or progeny production (e.g., *SI Appendix*, Fig. S3 vs. Fig. S4). Taken together, the transcript expression analysis revealed age-intrinsic differences in the epithelial response, revealing an age-adapted inflammatory response to SARS-CoV-2 infection that was elevated in adult compared to child BE.

## Discussion

In this study, we used human reconstituted BE to investigate the onset of infection and replication of SARS-CoV-2. BE are an important tissue because subsequent infection of the bronchial tissue determines whether a SARS-CoV-2 infection results in severe or mild respiratory illness, by controlling the spread into the lower respiratory tract. These features differentiate our approach from similar studies using primary respiratory cells from either upper airway (nasal, tracheal) (3, 26) or commercial sources (25) with undefined donor material. We followed the time course of infection for individual donors with imaging and RT-qPCR, showing that infections are detected as early as 1 dpi. The infection spread throughout the whole BE within 3 dpi to 4 dpi, and viral replication reached a plateau at around 4 dpi. We identified multiciliated cells as primary targets at infection onset, in agreement with previous studies (24, 26). Starting at 3 dpi, we observed large infected multinucleated syncytia forming between ciliated and basal cells. Formation of cell fusions in coronavirus-infected primary airway epithelia was previously reported (26, 46) but not systematically detected (24, 25, 32). Our observations are consistent with previous reports that infection of multiciliated cells with SARS-CoV-2 results in cilia loss and cell dedifferentiation (25, 26). The fusogenic potential of SARS-CoV-2 is now well described and involves the Spike protein and the ACE2 receptor (27, 47). A possible role for cell fusion could be facilitated cell-to-cell virus transmission, a mechanism recently reported for cultured cells (48). Syncytia formation was transient and reached a maximum at 4 dpi to sharply drop toward 7 dpi. The sharp drop in syncytia, but not in the number of overall infected cells, is consistent with our observation that syncytia were extruded into the lumen at the apical side of the epithelia. Those syncytia were anti–N positive, and EM analysis showed that they contained high amounts of virus trapped in a vesicular compartment, suggesting active progeny production. Importantly, we show that released syncytia and infected cells were a significant source of virus to infect target cells. Thus, cell- and syncytia-associated virus may help spread large and compact amounts of viruses into the upper respiratory tract, from which cell-associated viruses could either descend into the lower respiratory tract or be exhaled into the environment. Interestingly, patients who have succumbed to COVID-19 can present abnormal syncytia formed by pneumocytes in the lower respiratory tract, suggesting that our BE model may mimic severe forms of COVID-19 (13, 49, 50). Cell-associated virus release also relays to high detection of SARS-CoV-2 in sputum and its transmission by droplets in hospitalized patients (18). Massive but transient production of syncytia is reflected in another report showing that virus production in a primary airway epithelium is cyclic, with peaks of virus release every 7 d to 10 d (24). A periodicity or variability in the quantity of virus released from infected tissue thus may affect contagion and could provide an explanation for the phenomenon of “superspreader,” frequently suggested based on epidemiological data (51). Furthermore, we also observed the loss of cilia in many of the syncytia, which could result in poor mucociliary clearance that impedes the evacuation of viral particles and pathogens. Taken together, our findings are in accordance with previous findings but highlight syncytium formation as an important mechanism to understand the spreading of SARS-CoV-2 and the physiopathology of BE infection (13, 27, 49, 50).

Strikingly, we found very different propagation kinetics of SARS-CoV-2 in some BE when we included BE from children in our analysis. While permissive BE from children and adults showed similar infection kinetics, about one-third of all analyzed child BE (3/10) and a few adult BE (3/25) yielded very low overall viral production following BE inoculation. In agreement with the virus quantification, we observed very few infected cells in the BE that were included for time course analysis. Rather than rapidly spreading throughout the entire BE, the infected cells first formed a local cluster or foci of infected cells. From these foci, the infection slowly spread into the surrounding bystander cells. Yet, syncytia formation was also occasionally observed, at least in one restricted BE, suggesting that the fusion of basal cells with multiciliated cells is not inhibited, but infection spread is delayed.

The obvious difference in susceptibility to SARS-CoV-2 infection was mostly observed for BE from children. This correlates with epidemiological studies highlighting their reduced infection rate and lower death rate compared to adults/the elderly (23, 52). A recent study using nasal swab–derived BE also showed differences in the susceptibility to SARS-CoV-2 infection between adults and children (32). The reason for this intrinsic age difference is unknown. We show that, in our model, nonpermissive BE have a quicker release of IFN-λ in response to SARS-CoV-2 infection, starting as soon as 1 dpi, preempting virus spread, whereas permissive BE exhibit significant level of IFN-λ only at 3 dpi when virus spread has commenced. In contrast, type I IFN (i.e., α and β) were not detected either in the supernatant or in the transcript analysis. Type III IFN is recognized as a main driver of mucosal antiviral immunity (53). We confirm the importance of IFN-λ in infection control by showing that exogenous application can protect permissive BE from infection, while removing IFN-λ genes can promote infection. Recent studies show that SARS-CoV-2 blocks the IFN response by targeting the RIG-I/MDA-5 pathway (54, 55), suggesting that an initial race between virus replication and epithelial response determines the outcome of the infection. According to our data, the IFN response in nonpermissive BE could be faster, and an antiviral state is probably induced throughout the BE to slow down virus spread. Whether this is the result of better virus sensing by an unknown mechanism or whether SARS-CoV-2 is less able to counteract the IFN response are important questions to be addressed. Age-intrinsic properties of the epithelia might also play a role in the infection dynamics, because infection restriction was more commonly (but not exclusively) observed in child BE. We observed a strong proinflammatory response to SARS-CoV-2 infection in adult BE that was activated from 2 dpi and persisted for the time course. In contrast, the inflammatory response was much more attenuated in child BE compared to adults. This obvious difference did not affect virus spread or progeny production, but hyperinflammatory response is a hallmark of severe COVID-19 (15, 16). Our observation could thus be of importance to understand why children are less prone to severe COVID-19. Besides, age-related susceptibility to infection for BE has been reported for other respiratory pathogens, including respiratory viruses such as Rhinovirus-C, Adenovirus, and RSV (Respiratory Syncytial Virus) (56–58) but also fungi (*Aspergillus fumigatus*) (59) and bacteria (*Haemophilus influenzae*) (60).

Taken together, our data identify age-intrinsic differences in the inflammatory epithelial response to SARS-CoV-2 infection and identify the type III IFN response as a central contributor to SARS-CoV-2 resistance in BE. Importantly, partial resistance can be conferred to permissive BE when IFN-λ is applied in a timely manner.

## Materials and Methods

### Viruses and Cell Lines.

Vero E6 cells were maintained in Dulbecco’s modified Eagle’s medium (DMEM, Gibco) supplemented with 10% fetal calf serum and gentamicin (50 µg/mL) at 37 °C in a humidified CO_2_ incubator. The SARS-CoV-2 strain BetaCoV/France/IDF0372/2020 was supplied by the National Reference Centre for Respiratory Viruses hosted by Pasteur Institute through the European Virus Archive goes Global (EVAg platform). Agreement to work with infectious SARS-CoV-2 was obtained, and all work with infectious SARS-CoV-2 was performed in a Class II Biosafety Cabinet under BSL-3 conditions at the UB’L3 facility (TransBioMed core, Bordeaux).

### Viral Production.

The SARS-CoV-2 strain was produced by infecting Vero E6 cells at a multiplicity of infection of 1,200 PFU, then incubating the cells at 37 °C in a humidified CO_2_ incubator until appearance of a cytopathic effect (around 72 h). The culture supernatant was clarified by centrifugation (5 min at 1,500 rpm), and aliquots were stored at −80 °C. All viral stocks were sequenced to confirm that no mutation was selected during cultures, using Sanger to determine the full-length Spike sequence (61) as well as Oxford nanopore technology for whole genome sequencing, Stock titers were determined by adding serial dilutions to 2 × 10^4^ Vero E6 cells in supplemented DMEM in a 96-well plate. Eight replicates were performed. Plates were incubated at 37 °C and examined for cytopathic effect. Quantification of cytopathic effect was determined using the Cell tox green cytotoxicity assay (Promega) according to manufacturer instructions, and a Victor Nivo reader (Perkin-Elmer). The TCID_50_ per milliliter was calculated according to the method of Reed and Muench (62). The viral titer expressed in PFU per milliliter was mathematically converted from the TCID_50_ per milliliter determination using the Poisson equation as follow: PFU/mL = −ln 0.5 * TCID_50_/mL.

### Culture of Primary BE and Ethics Statement.

The BE cell culture was established from bronchial brushings or lung resection performed between the third and fifth bronchial generation from patients undergoing elective surgery as previously described (38). BE explants were cultured using PneumaCult Ex medium (Stemcell) for expansion of basal epithelial cells at 37 °C in 5% CO_2_. Then, 10^5^ basal cells were grown on cell culture inserts (Corning) within the air–liquid interface for 21 d using PneumaCult ALI medium (Stemcell). Such a culture allows the differentiation into pseudostratified mucociliary airway epithelia composed of ciliated cells, goblet cells, club cells, and basal cells. The complete differentiation was assessed by the capacity of cilia to beat and mucus production under a light microscope. According to the French law and the MR004 regulation, patients or children’s parents received an information form, allowing them to refuse the use of their surgical samples for research. TUBE is a collection of human bronchial tissue obtained from surgery and is sponsored by the University Hospital of Bordeaux, which includes its own local ethic committee (CHUBX 2020/54). All samples were deidentified prior to their use in this study.

### Infection of Epithelia.

Prior to infection, BE were washed three times with PBS to remove mucus, and basal ALI medium was exchanged with 500 µL of fresh medium. The inoculum containing 1,200 PFU of virus or medium-only controls was added to the apical surface to a final volume of 100 µL. Viral supernatant was removed after 1 h incubation at 37 °C and washed 3 times with PBS, and infection was followed for the indicated time points. Viral production was then quantified by RT-qPCR using three consecutively collected apical washes of 100 µL of PBS.

### Quantification of SARS-CoV-2 RNA by RT-qPCR.

For quantification of viral RNA by RT-qPCR, total RNA was isolated using the High Pure Viral RNA kit (Roche) according to the manufacturer’s instructions. Viral RNA was quantified using GoTaq 1-Step RT-qPCR kit (Promega). SARS-CoV-2 N gene RNA was amplified using forward (Ngene F cgcaacagttcaagaaattc 28844 to 28864) and reverse (Ngene R ccagacattttgctctcaagc 28960 to 28981) primers. Copy numbers were calculated from a standard curve produced with serial 10-fold dilutions of SARS-CoV-2-RNA. The amplification program began with the RT step for 15 min at 50 °C, then the denaturation step for 10 min at 95 °C, and 10 s at 95 °C, 10 s at 60 °C, and 10 s at 72 °C (40 cycles). The melting curve was obtained by temperature increment 0.5 °C/s from 60 °C to 95 °C.

### IFN Enzyme-Linked Immunosorbent Assay and IFN Treatment.

Human IL-29/IL-28B (IFN-λ 1/3) concentration in SARS-CoV-2–infected epithelium basal media was quantified using enzyme-linked immunosorbent assay (ELISA) techniques following the manufacturer’s recommendations (R&D systems); 100 µL of media was used for each point. Two prior infection BE were treated with either 5 ng or 50 ng of IFN-λ3 (R&D systems ref 5259-IL-025) added in the basal medium. Cells were then infected with 1,200 PFU as previously described. Four dpi, viral production was measured by RT-qPCR, and cells were fixed for immunostaining processing.

### Transcriptomic Analysis (NanoString).

Total RNA extracted from BE was used for 255 gene expression inflammation panel assays using NanoString technology. Multiplexed target enrichment was then performed with gene-specific primers from the “nCounter Inflammation panel” by following the manufacturer’s recommendations (see *SI Appendix* for details).

### Imaging and Image Analysis.

For antigen detection, BE were processed for IF analysis or EM as detailed in *SI Appendix*. Mounted samples were subsequently examined on an epifluorescence microscope (Leica inverted DMi6000 widefield microscope) at low magnification and at high magnification on a SP8 confocal microscope (Leica Microsystems at the Bordeaux Imagery Center). Image processing was done using Image J software or with Leica LAS-X software with task-adapted protocols (see *SI Appendix* for details). For EM analysis, grids were examined with a Transmission Electron Microscope (H7650, Hitachi) at 80 kV.

## Supplementary Material

Supplementary File

Supplementary File

Supplementary File

Supplementary File

Supplementary File

Supplementary File

Supplementary File

Supplementary File

Supplementary File

Supplementary File

Supplementary File

Supplementary File

Supplementary File

Supplementary File

Supplementary File

## Data Availability

All study data are made available and included in the article and/or supporting information.
