# Supplementary material for: Bronchial epithelia from adults and children: SARS-CoV-2 spread via syncytia formation and type III interferon infectivity restriction
Source: Proc Natl Acad Sci U S A. 2022 Jun 24;119(28):e2202370119. doi: 10.1073/pnas.2202370119 (PMC9651868; doi:10.1073/pnas.2202370119)
Supplement: Supplementary File [file pnas.2202370119.sapp.pdf]

Bronchial epithelia from adult and children: SARS-CoV-2 spread via syncytia formation and Type-III interferon infectivity restriction

Guillaume Beucher[a*], Marie-Lise Blondot[a*], Alexis Celle[b*], Noémie Pied[a], Patricia Recordon-Pinson[a], Pauline Esteves[b], Muriel Faure[a], Mathieu Métifiot[a], Sabrina Lacomme[c], Denis Dacheux[a,d], Derrick R. Robinson[a], Gernot Längst[e], Fabien Beaufils[b,f], Marie-Edith Lafon[a,g], Patrick Berger[b,h], Marc Landry[c,i], Denis Malvy[j], Thomas Trian[b#], Marie-Line Andreola[a#] and Harald Wodrich[a#]

[a]UMR 5234, Microbiologie Fondamentale et Pathogénicité, Centre national de la recherche scientifique (CNRS), Université de Bordeaux, 33076 Bordeaux, France

[b]U1045, Centre de Recherche Cardio-thoracique de Bordeaux, Institut national de la santé et de la recherche médicale (INSERM), Université de Bordeaux, 33076 Bordeaux, France

[c]UMS 3420, Bordeaux Imaging Center (BIC), Université de Bordeaux, 33076 Bordeaux, France

[d]Bordeaux INP, Microbiologie Fondamentale et Pathogénicité, UMR 5234, 33076 Bordeaux, France

[e]Biochemistry Center Regensburg, Universität Regensburg, 93053 Regensburg, Germany

[f]Service de pédiatrie médicale, Centre Hospitalier Universitaire (CHU) de Bordeaux, 33076 Bordeaux, France

[g]Service de Virologie et l'unité de surveillance biologique, Centre Hospitalier Universitaire (CHU) de Bordeaux, 33076 Bordeaux, France

[h]Service d'exploration fonctionnelle respiratoire, Centre d'investigation clinique (CIC) 1401, Centre Hospitalier Universitaire (CHU) de Bordeaux, 33076 Bordeaux, France

[i]UMR 5293, Institute of Neurodegenerative Diseases, Institut interdisciplinaire de neurosciences (IINS), Centre national de la recherche scientifique (CNRS), Institut national de la santé et de la recherche médicale (INSERM), Université de Bordeaux, 33076 Bordeaux, France

[j]Department for infectious and tropical diseases, Centre Hospitalier Universitaire (CHU) de Bordeaux, 33076 Bordeaux, France

Supplemental information (SI)

Supplemental methods (Imaging)

Immunofluorescence detection, antibodies and confocal microscopy.

For antigen detection, BE were washed repeatedly with PBS to remove mucus then fixed with 4% paraformaldehyde for 30min using complete insert immersion. Epithelia were then washed in PBS and

permeabilized with 0.5% TritonX-100 in PBS for 10min at room temperature and washed again before being blocked in IF buffer (PBS containing 10% SVF and 0.05% saponin) for 1h at room temperature. Primary antibody was diluted in IF buffer and applied to inserts for 1h at room temperature. Samples were washed three times under agitation with PBS and incubated with secondary antibody, fluorescently labeled phalloidin to stain the actin cytoskeleton and 2µg/mL of DAPI (4',6-diamidino-2-phenylindole), diluted in IF buffer and incubated for 2h at room temperature. Insert were then washed extensively in PBS, desalted in H_2O miliQ and rinsed in 100% Ethanol and air-dried. Membranes were then removed from inserts and mounted in DAKO Fluorescence Mounting Medium prior to microscopy analysis. Mounted samples were subsequently examined on an epifluorescence microscope (Leica inverted DMi6000 widefield microscope) at low magnification (5x, 20x) for kinetic studies. For higher magnification the imaging was performed on a SP8 confocal microscope (Leica Microsystems at the Bordeaux Imaging Center) using maximal pixel resolution at 10x, 20x or 63x using respectively, 2.99µm , 0.55µm and 0.21µm Z-stacks resolution. The following primary antibodies and IF dilutions were used in this study: mouse monoclonal Ab anti-SARS-CoV-2-N clone 3G9 (this study, 1:500), rabbit monoclonal Ab anti-human Cytokeratin 5 (Abcam, ab52635, 1:200), guinea pig polyclonal Ab anti-Cytokeratin 5 (Origene, BP5006, 1:200), rabbit monoclonal Ab anti-human Acetyl alpha tubulin (Cell Signaling, D20G3, 1:200), mouse monoclonal Ab anti-Alpha-tubulin-acetyl K40 (Abcam, ab24610), rabbit polyclonal Ab anti-human ACE2 (Abcam, ab15348, 1:50), rabbit monoclonal Ab anti-human-Mucin 5AC (Abcam, ab3649, 1:200), mouse monoclonal Ab anti-human-Mucin 5AC (Abcam, ab2649, 1:200). The following secondary antibodies were used in this study: cross absorbed Donkey anti-mouse Alexa Fluor 488 or 647 (Invitrogen, A212020/A31573, 1:300), cross absorbed Donkey anti-rabbit Alexa Fluor 594 (Invitrogen, A31573, 1:300) and cross absorbed Donkey anti-guinea pig Alexa Fluor 594 (Invitrogen, A11076, 1:300) as well as Texas Red labeled phalloidin (Invitrogen, 1:500).

Monoclonal anti-nucleocapsid (N) antibodies and ethics statement

Monoclonal antibodies were raised against bacterially expressed and purified SARS-CoV-2 N protein in 3 mice using the protocol previously described (59). Hybridomas were cloned by limiting dilution and screened by immunofluorescence on infected Vero E6 cells. Clone 3G9 was retained for this study and antibody was affinity purified from hybridoma supernatant prior to use. Mice experiments have been performed in the conventional animal facilities of the University of Bordeaux (France) (approval number of B-33-036-917), with the approval of institutional guidelines determined by the local Ethical Committee of the University of Bordeaux and in conformity with the Ministry for Higher Education and Research and the French Committee of Genetic Engineering (approval number n °17621 -V5-2018112201234223).

Image analysis and quantification.

Full epithelia overviews were acquired with Leica LAS-X software in spiral mosaic mode and three-dimensional reconstructions were done with Leica LAS-X software in 3D-viewer mode. Image processing was done using Image J software with task adapted macros. Signals of interest were quantified using a semi-automatic macro. In general, Z-projections of different focal planes were generated and regions of interest (ROI) were manually inserted. Signal of interest was quantified automatically in each ROI, with appropriate predefined threshold and sizing for each condition. Quantifications were performed to measure either number or size of signal of interest. Obtained values are represented either as absolute number or as normalized values (as indicated).

<u>Time course of infection (Fig.1E, 1F, 4D, 4E)</u>: To determine the number and size of N positive cells the entire epithelial surface was recorded at 10x magnification for each time point, for each donor and processed for image analysis. Z-projections of different focal planes of the entire epithelium were generated and several regions of interest (ROI) were designed to delimit the area of analysis and applied to all epithelia. A threshold was then applied to the projection to obtain a mask representing N positive cells used to calculate number and size of N positive signals using the "analyze particles" function in ImageJ.

<u>IFN experiment (Fig. 5C, 5G)</u>: To determine the infected surface in presence or absence of IFN treatment the entire epithelial surface was recorded at 10x magnification for each condition, for each donor and processed for image analysis. Z-projections of the entire epithelium were generated and determined as ROI. The infected area was determined by the N-signal and calculated as percentage of infected area or as normalized value to the non-treated control.

<u>Colocalisation analysis between N and marker of either basal, goblet or ciliated cells (Fig. 1C, 2C, S5B):</u> To quantify colocalisation between N positive cells (infected cells) and either cytokeratin 5 (Cytk5, basal cells), mucin 5A (goblet cells) or acetylated tubulin (multi ciliated cells) 10x magnification images or a 63x magnification images were acquired and processed for image analysis. Z-projections of different focal planes were generated and regions of interest (ROI) were designed to delimit the area of analysis. A threshold was then applied to the projection of both N positive cells and Cytk5 (or tubulin) positive cells in order to obtain respective masks. Masks were analyzed using the "colocalization" function in ImageJ to calculate the number of double positive cells (N and Cytk5). Obtained values were normalized by either total number of N or total number of Cytk5 (or tubulin or mucin) positive cells to calculate the percentage of basal cells (or ciliated/goblet cells) that are infected.

Syncytia (nuclei) quantification (Fig. 2B): Randomly obtained syncytia-like structures (based on the N-signal) were taken at 63x magnification and individual planes were analyzed (*ie* no Z-projection to avoid false inclusion of nuclei). The N signal was used as a marker of infected cells and used as ROI followed by manual quantification of nuclei in the ROI analyzing several focal planes.

Electron microscopy

For electron microscopy, BE were first washed in physiological serum and then fixed with 2.5% (v/v) glutaraldehyde and 2% (v/v) paraformaldehyde in 0.1M phosphate buffer (pH 7.4) during 2h minimum at room temperature (RT). Then samples were washed in 0.1M phosphate buffer and post-fixed in 1% (v/v) osmium tetroxide in phosphate buffer 0.1 M during 2h, in the dark, at RT, then washing in water and dehydrated through a series of graded ethanol and embedded in a mixture of pure ethanol and epoxy resin (Epon 812; Delta Microscopy, Toulouse, France) 50/50 (v/v) during 2 hours and then in 100% resin overnight at RT. The polymerization of the resin was carried out over a period between 24-48 hours at 60°C. Samples were then sectioned using a diamond knife (Diatome, Biel-Bienne, Switzerland) on an ultramicrotome (EM UCT, Leica Microsystems, Vienna, Austria). Ultrathin sections (70 nm) were picked up on copper grids and then stained with uranyless and lead citrate. Grids were examined with a Transmission Electron Microscope (H7650, Hitachi, Tokyo, Japan) at 80kV.

CRISPR/Cas9 gene targeting

The protocol was adapted from Kyung Duk Koh *et al*. (Koh et al., 2020, Am J Respir Cell Mol Biol). Single guide sgRNA sequences against IFNλ2 (C*A*G*GCCCCAACGACUCACAC, C*A*G*GCCCCAACGACUCACAC°) and purified Cas9 were purchased from Synthego. Respective sgRNAs were suspended in TE buffer and combined with Cas9 and incubated at room temperature for 10 minutes before electroporation. For gene targeting, primary bronchial epithelial cells were expanded to reach 80% confluence and harvested for electroporation. A total of 150,000 cells was suspended in 20 µl of P3 Primary Cell Nucleofector Solution (Lonza) and mixed with the sgRNAs/Cas9 solutions before electroporation (4D-Nucleofector System; Lonza; program DC-100). For control conditions, bronchial epithelial cells were electroporated with Cas9 in absence of sgRNAs. Electroporated bronchial epithelial cells were seeded on human placental collagen-coated 6-well dishes for recovery during 3 days before a second round of electroporation in presence of sgRNAs/Cas9 mix was performed followed by another recovery period. Then, gene targeted and control bronchial epithelial cells respectively were cultured in ALI medium for 21 days for epithelial differentiation. Gene editing efficiency was verified using PCR analyses of the targeted gene with primers encompassing the gene deleted part.

Multiplex gene expression analysis

BE cells from adults and children were lysed by adding 200 µl of lysis buffer RLT according to manufacturer's instruction (AllPrep® DNA/RNA/Protein Mini Kit, Qiagen, Hilden, Germany) at different time point post infection with SARS-CoV-2 infection. mRNA extracts were stored at -80°C and send to PARS-I (Plateforme Analytique de Recherche en Santé-Immunologie, Dr. Isabelle Pellegrin, Bordeaux, France) to be assessed with nCounter® FLEX using the inflammation panel (nanoString, Seattle, WA). The list of genes included in the inflammation panel are listed in the supplemental table S1. Analysis was performed with nSolver™ Analysis software to compare differential RNA expression within the epithelium in the different conditions. Row data of differential expression in adults and children at 1, 2, 4 and 7 dpi compared with non-infected control BE are presented in the supplemental table S2.

Supplemental data

Supplemental figures (Fig. S1-S5), movies (Movie S1- S10) and tables (table S1 and S2)

Supplemental Figure 1

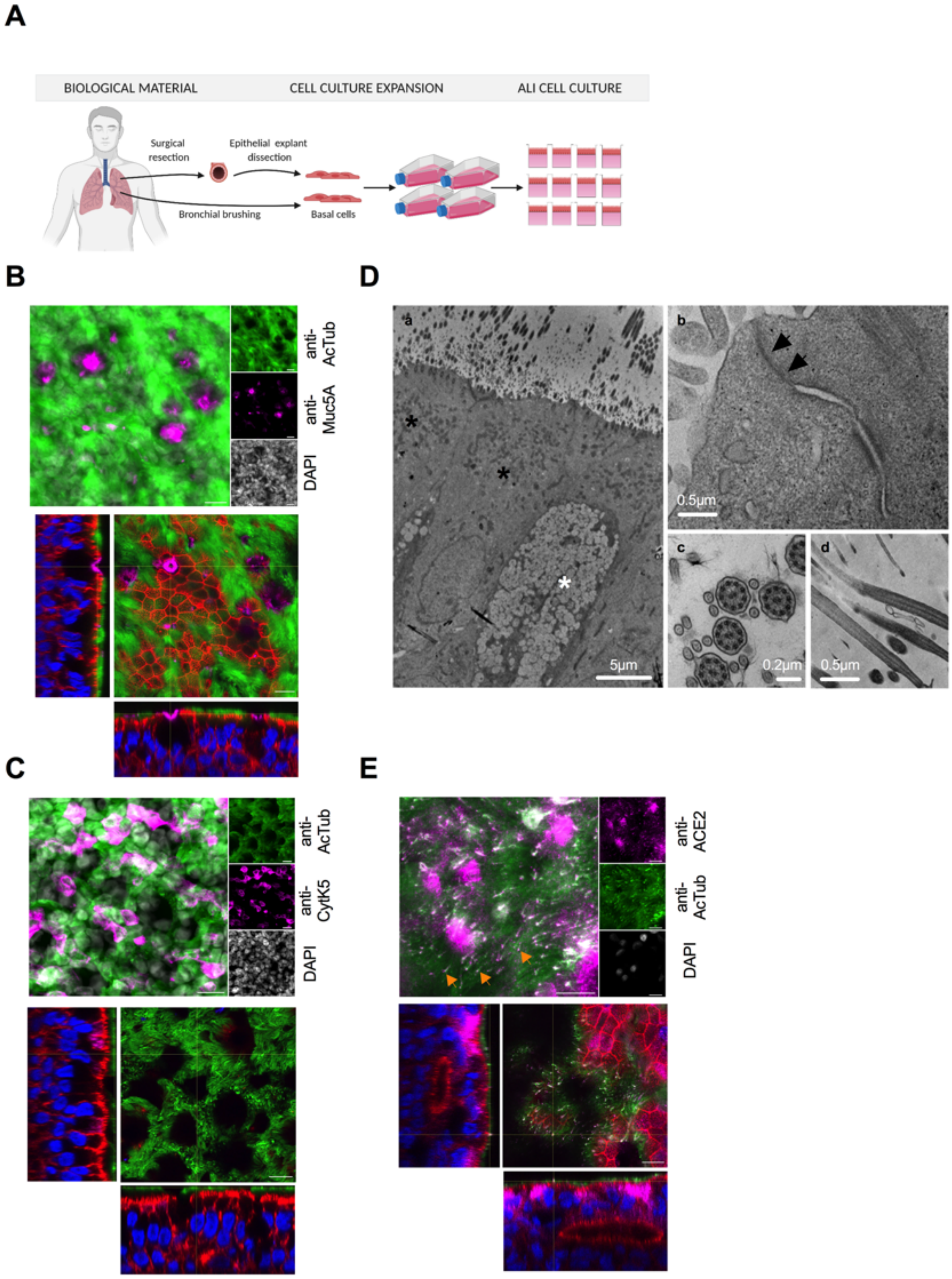

Figure S1. Characterization of BE. A: Schematic overview of BE generation. Basal cells extracted from surgical dissection or bronchial brushing were expanded and differentiated at the air-liquid interface. B: Differentiated BE were stained with anti-acetylated tubulin to identify ciliated epithelia cells (green signal) or anti-mucin 5A to detect goblet cells (pink signal) and counterstained with DAPI (grey in top image, blue in bottom image). Top image shows a Z-projection, the bottom image shows an individual Z-section counterstained with phalloidin to detect the cell morphology via the actin cell cortex (red signal). Scale bar is 10µm. Note that ciliated cells are located to the apical side (see movie S1 for 3D). C: As in B but the differentiated BE was stained with anti-acetylated tubulin (green signal) or anti-cytokeratin 5 to detect basal cells (pink signal) and counterstained with DAPI (grey in top image, blue in bottom image). Scale bar is 10µm. (see movie S2 for 3D). D: Electron microscopy of fully differentiated BE. The overview (a) shows ciliated epithelia cells (black asterisk) and goblet cells (white asterisk). The magnified images show tight junctions (b) marked by arrows and cilia either as cross-section (c) or longitudinal section (d). Scale bars are indicated. E: As in B but the differentiated BE was stained with anti-acetylated tubulin (green signal) or anti-ACE2 to detect the SARS-CoV-2 receptor (pink signal) and counterstained with DAPI (grey in top image, blue in bottom image). Note that arrows point at individual cilia with ACE2 signal. Scale bar is 10µm. (see movie S2 for 3D).

Supplemental Figure 2

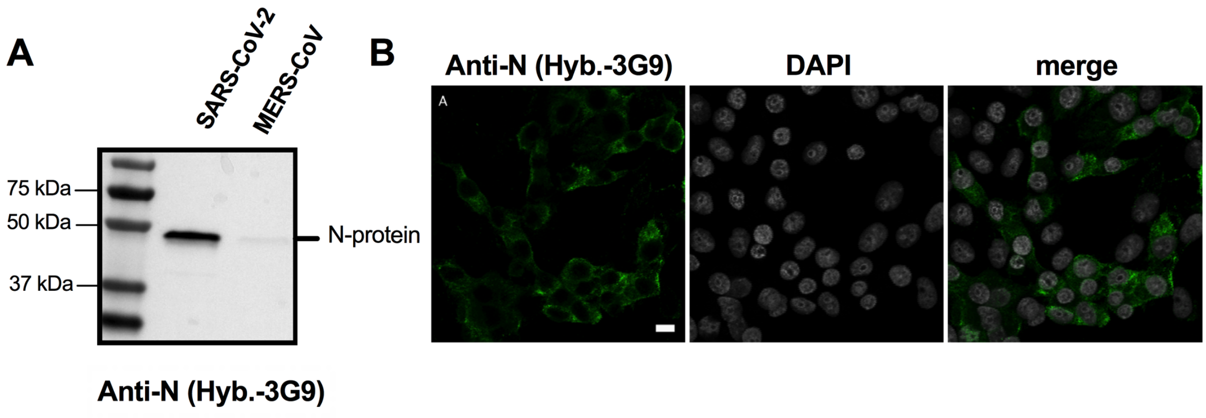

Figure S2. Characterization of monoclonal anti-SARS-CoV-2-N antibody (clone 3G9). A: Western blot analysis of recombinant bacterially purified SARS-CoV-2-N (100 ng, left lane) vs. MERS-CoV-N (100 ng, right lane). B: Detection of infected Vero E6 cells. Cells were infected for 24h with SARS-CoV-2, fixed and stained with monoclonal antibody to SARS-CoV-2-N (hybridoma 3G9).

Supplemental Figure 3

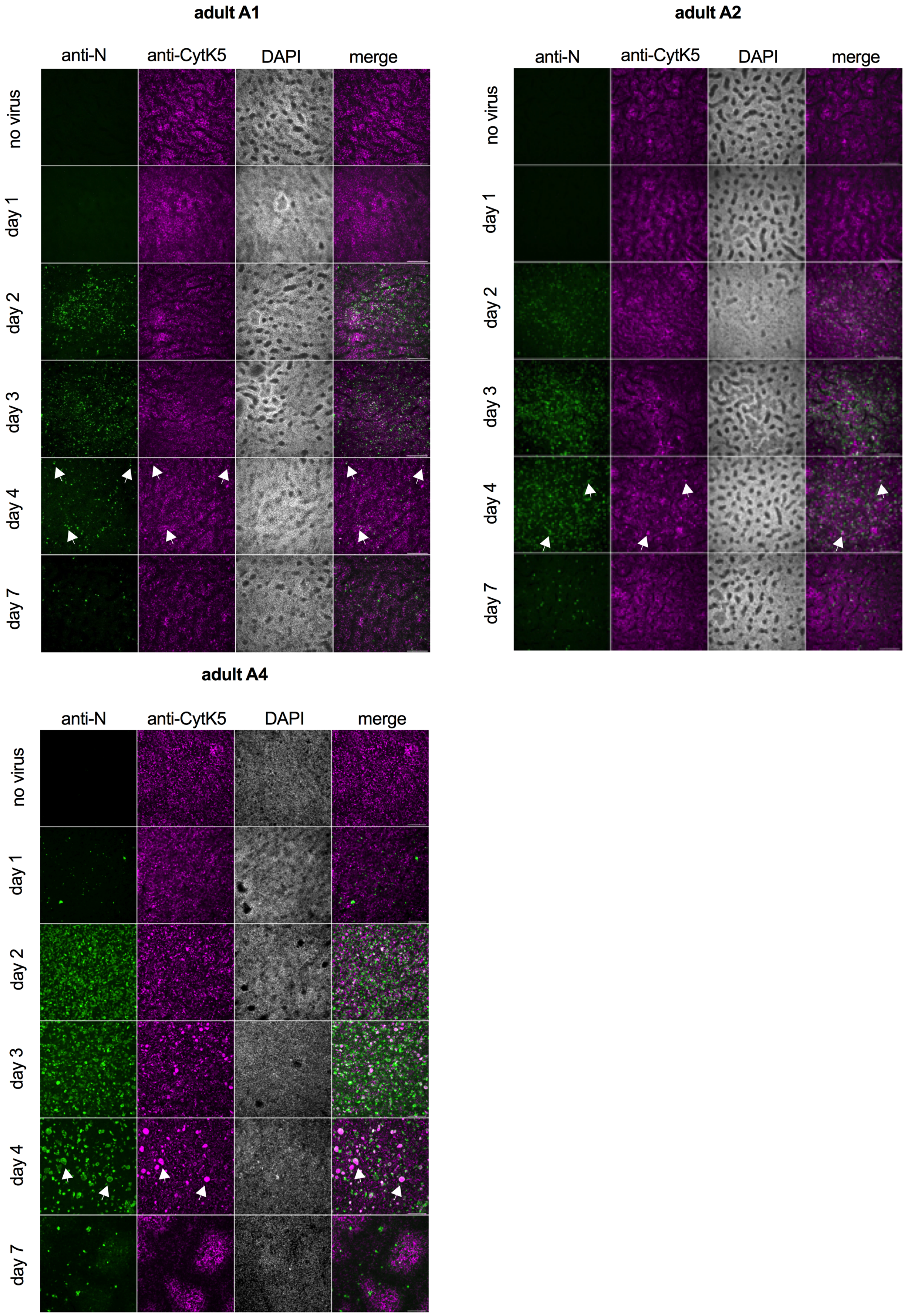

Figure S3. SARS-CoV-2 infection kinetic of BE. A: Representative widefield microscopy images of BE from adult donors (A1 top left, A2 top right and A4 bottom left) at low resolution. BE were fixed at day 1, 2, 3, 4, 7 as indicated to the left of each row, non-infected controls were also fixed at day 7. BE were stained with anti-N antibodies to detect infected cells (green signal first column), anti-cytokeratin 5 to detect basal cells (magenta signal, second column) and counterstained with DAPI (grey signal, third column) and merged (fourth column). Large specific signals in all channels are apparent on day four (white arrows). Scale bar is 50µm.

Supplemental Figure 4

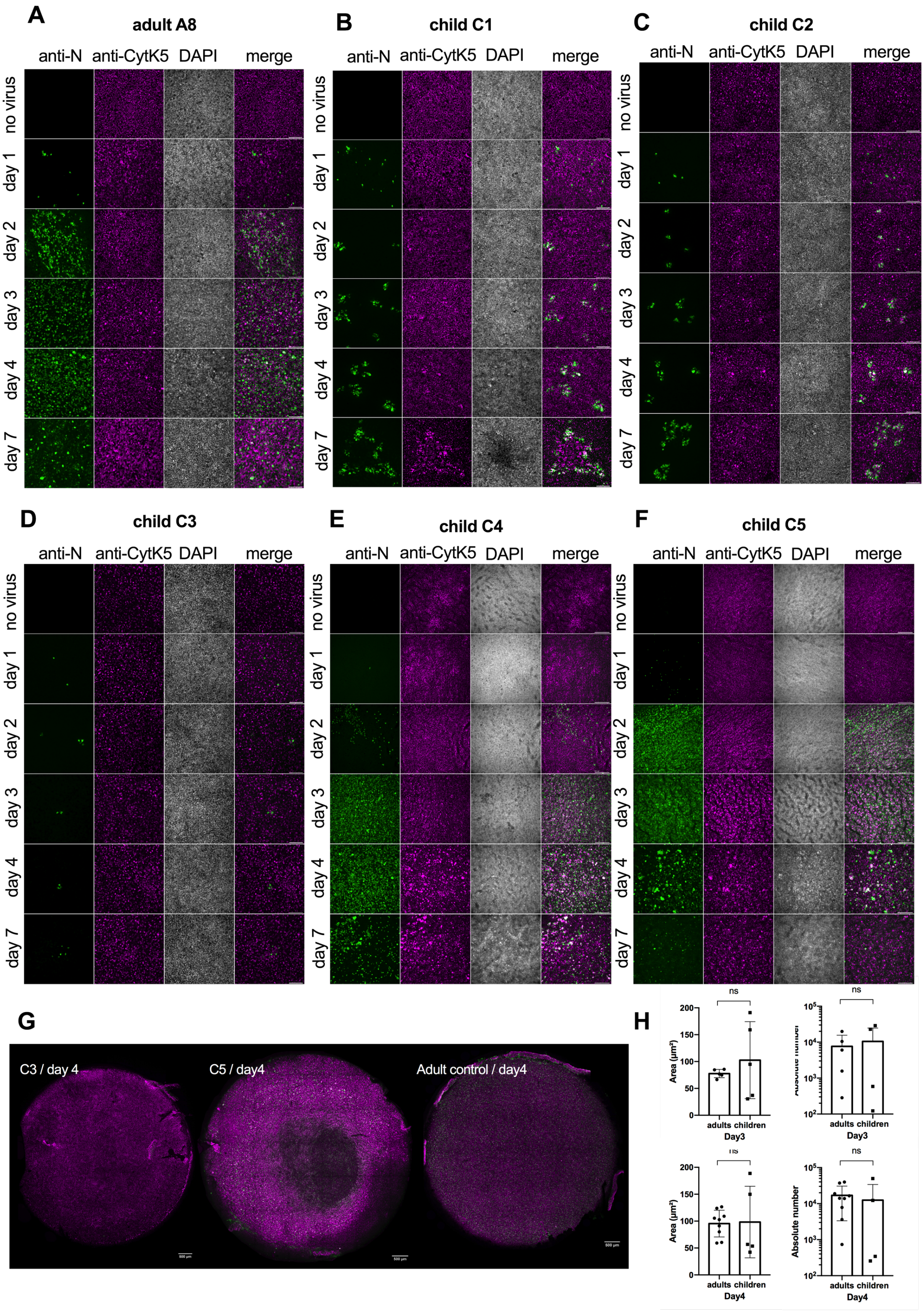

Figure S4. SARS-CoV-2 BE infection kinetic from children and adult donors. A-H: Representative widefield microscopy images of BE from one adult donor (A6 top left), three infection restricted children (C1-C3, top middle and right, bottom left) and two children permissive to infection (C4 and C5, bottom middle and right). BE were fixed at day 1, 2, 3, 4, 7 as indicated to the left of each row, non-infected controls were also fixed at day 7. BE were stained with anti-N antibodies to detect infected cells (green signal first column), anti-cytokeratin 5 to detect basal cells (magenta signal, second column) and counterstained with DAPI (grey signal, third column) and a merge of the first two signals (fourth column). Note the slow virus spread in the non-permissive children epithelia. Scale bar is 50µm. G: Entire BE overviews for non-permissive donor C3 and permissive donor C5 and the adult control. Scale bar is 500µm H: Comparison between adult and child BE in terms of number and size of infected cells at 3 and 4 dpi. Data include all available BE from this study for which the respective data could be collected. Samples include for day 3 adults A1-A4 and children C1-C5 and for day 4, A1-7, A11/12 and children C1-C5.

Supplemental Figure 5

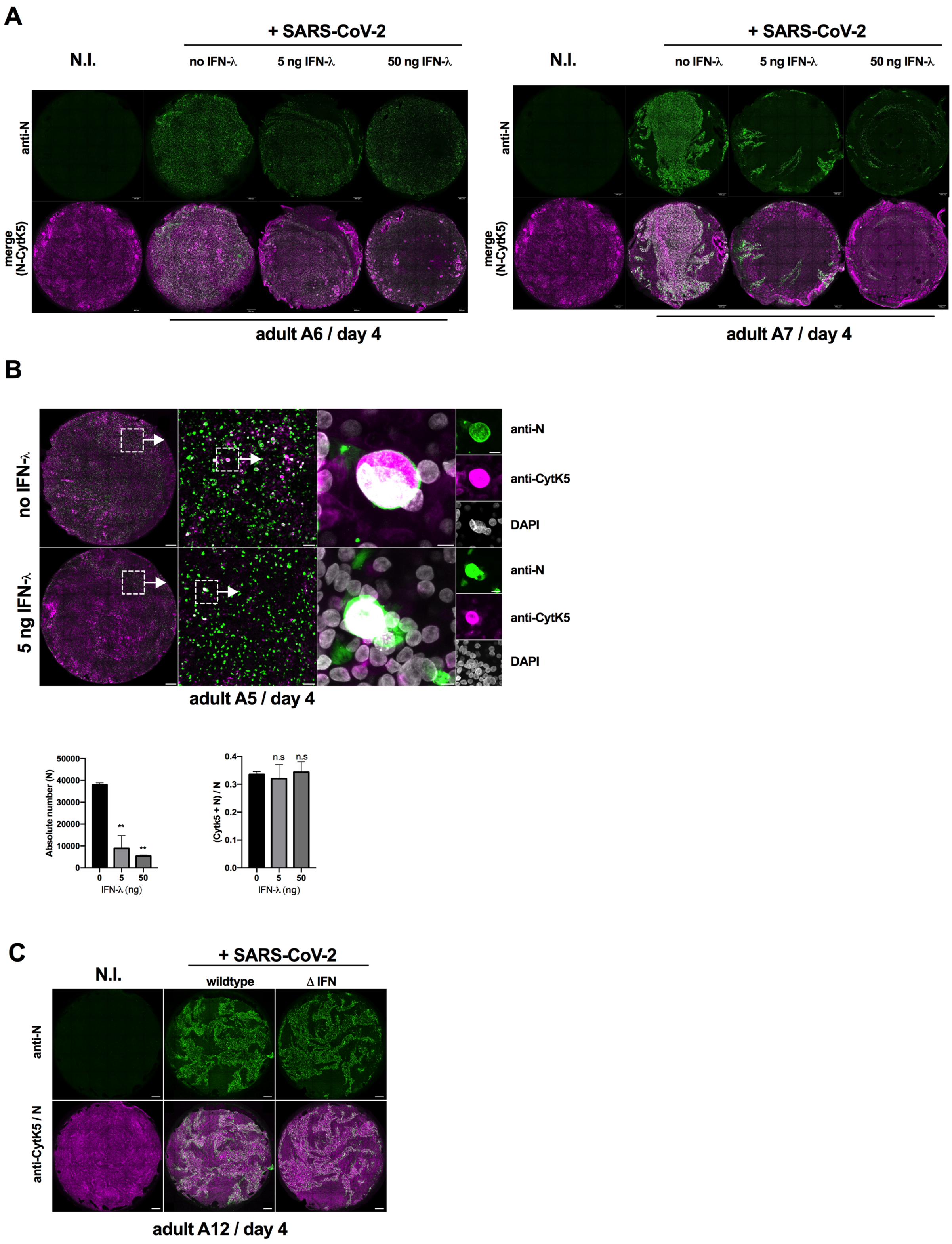

Figure S5. BE Infection with IFN-λ. A: BE Infection following IFN-λ treatment (shown on top). Entire BEs for donor A6 (left panel) and donor A7 (right panel) are shown at 4 dpi stained with anti-N antibodies to detect infected cells (green signal) and anti-cytokeratin 5 marking basal cells (magenta signal). Please note that the same non-infected control was used for both panels corresponding to main figure 5B B: Syncytia formation in IFN-λ treated BEs. Entire BEs for donor A5 with (bottom) and without (top) IFN-λ treatment are shown at 4 dpi stained as in (A). Boxed areas show BE magnifications (middle column) and individual syncytia (right column). Individual channels as indicated to the right. The graphs show quantification of total number of infected cells per BE (left panel) and N plus cytokeratin 5 double positive cells (middle panel) or normalized for total infected cells (right panel). Results are presented as mean ± SEM for donor A5 and A7 and significance was calculated using one sided ANOVA. C: Entire BEs from donor A12 are shown at 4 dpi following IFN-λ gene deletion and donor control as indicated and stained as in (A) and (B).

Supplemental movie legends

Movie S1: Differentiated BE were stained with anti-acetylated tubulin to identify multi-ciliated epithelia cells (green signal) or anti-mucin 5A to detect goblet cells (pink signal) and counterstained with DAPI to stain cell nuclei (blue signal) and phalloidin to detect the cell morphology via the actin cell cortex (red signal). Movies show the three-dimensional (apical to basolateral) organization of the epithelium with individual focal planes taken every 0.3 µm.

Movie S2: Differentiated BE were stained with anti-acetylated tubulin to identify multi-ciliated epithelia cells (green signal) or anti-cytokeratin 5 to detect basal cells (pink signal) and counterstained with DAPI to stain cell nuclei (blue signal) and phalloidin to detect the cell morphology via the actin cell cortex (red signal). Movies show the three-dimensional (apical to basolateral) organization of the epithelium with individual focal planes taken every 0.21µm.

Movie S3: Differentiated BE were stained with anti-acetylated tubulin to identify multi-ciliated epithelia cells (green signal) or anti-ACE2 to detect the SARS-CoV-2 cell receptor (pink signal) and counterstained with DAPI to stain cell nuclei (blue signal) and phalloidin to detect the cell morphology via the actin cell cortex (red signal). Movies show the three-dimensional (apical to basolateral) organization of the epithelium with individual focal planes taken every 0.21µm. Note the localization of ACE2 in individual cilia.

Movie S4: Differentiated BE were infected with SARS-CoV-2 and stained 24h post-infection with anti-N (green signal) to identify infected cells and anti-mucin 5A to detect goblet cells (magenta signal) and counterstained with DAPI to stain cell nuclei (blue signal) and phalloidin to detect the cell morphology via the actin cell cortex (red signal). Movies show the three-dimensional (apical to basolateral) organization of the epithelium with individual focal planes taken every 0.21µm.

Movie S5: Differentiated BE were infected with SARS-CoV-2 and stained 24h post-infection with anti-N (green signal) to identify infected cells and anti-cytokeratin 5 to detect basal cells (magenta signal) and counterstained with DAPI to stain cell nuclei (blue signal) and phalloidin to detect the cell morphology via the actin cell cortex (red signal). Movies show the three-dimensional (apical to basolateral) organization of the epithelium with individual focal planes taken every 0.21µm.

Movie S6: Differentiated BE were infected with SARS-CoV-2 and stained 24h post-infection with anti-N (green signal) to identify infected cells and anti-acetylated tubulin to detect multi-ciliated cells (magenta signal) and counterstained with DAPI to stain cell nuclei (blue signal) and phalloidin to detect the cell morphology via the actin cell cortex (red signal). Movies show the three-dimensional (apical to basolateral) organization of the epithelium with individual focal planes taken every 0.21µm.

Movie S7: Differentiated BE were infected with SARS-CoV-2 and stained 4 dpi with anti-N to detect infected syncytia (green signal) and anti-cytokeratin 5 marking basal cells (magenta signal) and counterstained with DAPI to stain cell nuclei (blue signal) and phalloidin to detect the cell morphology via the actin cell cortex (red signal). Movies show the three-dimensional (apical to basolateral) organization of the epithelium with individual focal planes taken every 0.21µm. Note that syncytia are double positive for N and cytokeratin 5.

Movie S8: Differentiated BE were infected with SARS-CoV-2 and stained 4 dpi with anti-N to detect infected syncytia (green signal) and anti-acetylated tubulin marking multi-ciliated cells (magenta signal) and counterstained with DAPI to stain cell nuclei (blue signal) and phalloidin to detect the cell morphology via the actin cell cortex (red signal). Movies show the three-dimensional (apical to basolateral) organization of the epithelium with individual focal planes taken every 0.21µm. Note that only one of the syncytia is double positive for N and acetylated tubulin.

Movie S9: Double staining of differentiated adult BE infected with SARS-CoV-2 and stained 4 dpi. The movie shows the boxed area in the top view of figure 2F. The BE is stained with anti-N to detect infected syncytia (yellow signal), anti-acetylated tubulin marking multi-ciliated cells (cyan signal) and counterstained with DAPI to stain cell nuclei (blue signal). Movie shows the three-dimensional (apical to basolateral) organization of the epithelium with individual focal planes taken every 0.21µm.

Movie S10: Double staining of differentiated adult BE infected with SARS-CoV-2 and stained 4 dpi. The movie shows the boxed area in the top view of figure 2F. The BE is stained with anti-N to detect infected syncytia (yellow signal), anti-cytokeratin 5 to detect basal cells (magenta signal) and counterstained with DAPI to stain cell nuclei (blue signal). Movie shows the three-dimensional (apical to basolateral) organization of the epithelium with individual focal planes taken every 0.21µm.

Movie S11: Triple staining of differentiated adult BE infected with SARS-CoV-2 and stained 4 dpi. The movie shows the boxed area in the top view of figure 2F. The BE is stained with anti-N to detect infected syncytia (yellow signal), anti-acetylated tubulin marking multi-ciliated cells (cyan signal) and anti-cytokeratin 5 to detect basal cells (magenta signal) and counterstained with DAPI to stain cell nuclei (blue signal). Movie shows the three-dimensional (apical to basolateral) organization of the epithelium with individual focal planes taken every 0.21µm. Note that only the syncytia are triple positive for N, cytokeratin 5 and acetylated tubulin.

Movie S12: C: Differentiated BE from a child donor was infected with SARS-CoV-2 and stained 4 dpi with anti-N to detect infected syncytia (green signal) and anti-cytokeratin 5 marking basal cells (magenta signal) and counterstained with DAPI to stain cell nuclei (blue signal) and phalloidin to detect the cell morphology via the actin cell cortex (red signal). Movies show the three-dimensional (apical to basolateral) organization of the epithelium with individual focal planes taken every 0.21µm. Note that syncytia are double positive for N and cytokeratin 5 but smaller then syncytia from adults (compare with movie S7 and S8).

Supplemental Dataset 1 legend

List of genes included in the Nanostring inflammatory panel. Genes are listed in alphabetic number with associated accession number.

Supplemental Dataset 2 legend

Row data of differential expression between adults and children after Nsolver analysis. Data are presented as log2 fold differential expression and –log10 of the p-value calculated between adults and children for each detected target.
